# *TEX11* is mutated in infertile men with azoospermia and regulates genome-wide recombination rates in mouse

**DOI:** 10.15252/emmm.201404967

**Published:** 2015-07-01

**Authors:** Fang Yang, Sherman Silber, N Adrian Leu, Robert D Oates, Janet D Marszalek, Helen Skaletsky, Laura G Brown, Steve Rozen, David C Page, P Jeremy Wang

**Affiliations:** 1 Department of Biomedical Sciences, School of Veterinary Medicine, University of PennsylvaniaPhiladelphia, PA, USA; 2 Infertility Center of St. Louis, St. Luke’s HospitalSt. Louis, MO, USA; 3 Department of Urology, Boston University Medical CenterBoston, MA, USA; 4 Howard Hughes Medical Institute, Whitehead InstituteCambridge, MA, USA; 5 Duke-Nus Graduate Medical School SingaporeSingapore City, Singapore; 6 Department of Biology, Massachusetts Institute of TechnologyCambridge, MA, USA

**Keywords:** chromosomal synapsis, infertility, meiosis, recombination, X chromosome

## Abstract

Genome-wide recombination is essential for genome stability, evolution, and speciation. Mouse *Tex11*, an X-linked meiosis-specific gene, promotes meiotic recombination and chromosomal synapsis. Here, we report that *TEX11* is mutated in infertile men with non-obstructive azoospermia and that an analogous mutation in the mouse impairs meiosis. Genetic screening of a large cohort of idiopathic infertile men reveals that *TEX11* mutations, including frameshift and splicing acceptor site mutations, cause infertility in 1% of azoospermic men. Functional evaluation of three analogous human *TEX11* missense mutations in transgenic mouse models identified one mutation (V748A) as a potential infertility allele and found two mutations non-causative. In the mouse model, an intronless autosomal *Tex11* transgene functionally substitutes for the X-linked *Tex11* gene, providing genetic evidence for the X-to-autosomal retrotransposition evolution phenomenon. Furthermore, we find that TEX11 protein levels modulate genome-wide recombination rates in both sexes. These studies indicate that *TEX11* alleles affecting expression level or substituting single amino acids may contribute to variations in recombination rates between sexes and among individuals in humans.

## Introduction

Infertility, defined as the inability to conceive after a prolonged period, is a worldwide reproductive health problem, affecting men and women about equally (Hull *et al*, 1985; Matzuk & Lamb, 2002). The underlying causes are multifaceted, including physiological, environmental, social, and genetic factors; and studies in various model organisms have identified multiple molecular and genetic pathways that regulate fertility (de Rooij & de Boer, 2003; Matzuk & Lamb, 2008). In particular, mouse models have identified more than 400 genes that are specifically or preferentially involved in the regulation of fertility, facilitating genetic studies of infertility in humans (Matzuk & Lamb, 2008; Handel & Schimenti, 2010; Jamsai & O’Bryan, 2011). In humans, male infertility is a more clearly defined entity in cases of azoospermia or severe oligospermia (Hull *et al*, 1985; Silber, 2000). Known genetic causes of azoospermia in humans include Y chromosome deletion and chromosomal abnormalities such as Klinefelter syndrome (47, XXY); these account for ∼25% of spermatogenic failure in otherwise healthy men (Reijo *et al*, 1995, 1996; Van Assche *et al*, 1996). Therefore, the majority (∼75%) of cases of spermatogenic failure in humans are idiopathic, and the underlying causes are postulated to be genetic. However, to date, efforts to uncover point mutations in single genes that contribute to human spermatogenic failure have been largely unsuccessful (Matzuk & Lamb, 2008; Nuti & Krausz, 2008; Jamsai & O’Bryan, 2011).

Two major hurdles complicate the molecular genetic analysis of human male infertility. Traditionally, many disease-causing monogenic mutations have been identified through pedigree-based linkage analyses, demonstrating that the mutation co-segregates with a disease in multi-generation families and can therefore be deemed causative. This conventional approach is not applicable to the study of human male infertility, because infertile men lack biological offspring; it is therefore difficult to determine whether a mutation is causative. The second hurdle is that clinical and ethical considerations limit the availability of sufficient testis biopsy material from patients for further validations. Genome-wide association studies (GWAS) have identified risk loci for non-obstructive azoospermia in humans (Hu *et al*, 2011, 2014). While variants in a number of genes have been identified in infertile men, causality of these variants has not been definitively proven (Sun *et al*, 1999; Miyamoto *et al*, 2003; Stouffs *et al*, 2005a,b, 2006; Krausz *et al*, 2006; Rohozinski *et al*, 2006; Martinez *et al*, 2007; Luddi *et al*, 2009).

Genomic studies have revealed that germ cell-specific genes are not randomly distributed in the genome, and that, in particular, the unique hemizygous and transcriptional status of the X chromosome has shaped its germ cell-specific gene content (Wang *et al*, 2001; Khil *et al*, 2004; Namekawa *et al*, 2006; Turner *et al*, 2006; Mueller *et al*, 2008; Song *et al*, 2009). The mammalian X chromosome is enriched for germ cell-specific genes expressed during early spermatogenesis. A systematic genomic screen of mouse spermatogonia, which are diploid mitotic germ cells of the testis, identified dozens of genes that are expressed specifically in male germ cells, and nearly one-third of these genes map to the X chromosome, suggesting that genes encoded on the X chromosome play a preeminent role in early spermatogenesis (Wang *et al*, 2001). Genetic studies in mouse models have shown that three of these X-linked genes (*Tex11*, *Taf7 l*, and *Nxf2*) are important regulators of male fertility (Cheng *et al*, 2007; Yang *et al*, 2008; Pan *et al*, 2009; Zheng *et al*, 2010). As males are hemizygous for the X chromosome, mutations in single-copy X-linked genes cannot be compensated by a corresponding wild-type allele such as in heterozygous carriers of autosomal recessive mutations. Therefore, mutations in X-linked genes essential for fertility may represent a significant proportion of infertility-causing mutations in men.

*Tex11* is essential for male fertility in mice. Disruption of *Tex11* gene function causes meiotic arrest in males, resulting in azoospermia (Yang *et al*, 2008). Here, we report that the frequency of rare *TEX11* mutations is significantly elevated in azoospermic men, suggesting that *TEX11* is required for spermatogenesis in humans. In combination with analyses of genetically modified mice harboring *Tex11* mutations analogous to those in human, our results demonstrate that in ∼1% of azoospermic men, infertility is caused by mutations in a single X-linked gene—the *TEX11* gene. Furthermore, our studies show that meiotic progression requires a critical threshold level of TEX11 protein and, significantly, that genome-wide meiotic recombination rates in both sexes are sensitive to TEX11 levels.

## Results

### Frequent singleton *TEX11* mutations in men with spermatogenic failure

To evaluate the role of *TEX11* in human fertility, we screened genomic samples from 246 azoospermic men with spermatogenic failure (no sperm in semen) and from 175 controls that included men who had fathered children (*n* = 93) and men of unknown fertility selected to represent worldwide genetic diversity based on their Y-chromosomal haplotypes (*n* = 82). All the infertile patients selected were pre-screened for the lack of Y chromosome microdeletions. Sequencing of amplicons covering the *TEX11* exons and flanking intronic regions revealed 40 different sequence variants in *TEX11* in our cohorts (Table1 and Supplementary Table S1). Of these variants, 21 were singletons (observed in only one man; Table1), whereas 19 were observed in two or more men and thus are not likely to be associated with spermatogenic failure (Supplementary Table S1).

**Table 1:** Singleton sequence variants in *TEX11* found in infertile patients and controls

Position	Nucleotide change	Resultant change	Patient ID	Infertile males	Control males	
				AZ	Fertile	NIH diversity
				246^a^	93^a^	82^a^
Exon 6	349T→A	Missense mutation, W117R	WHT3150	1		
Exon 6	405C→T	Silent mutation	WHT3171	1		
Exon 7	424G→A	Missense mutation, V142I	WHT3417	1		
Exon 7	515A→G	Missense mutation, Q172R	WHT3500	1		
Exon 10	731C→T	Missense mutation, T244I	WHT2546	1		
Exon 16	1258Ins (TT)	Frameshift mutation; 1258GATG→TTGGTA	WHT3759	1		
Exon 26	2243T→C	Missense mutation, V748A	WHT2499	1		
Exon 27	2319T→C	Silent mutation	WHT2546	1		
Intron 3	−17T→C^b^	Intronic alteration	WHT3448	1		
Intron 5	−48G→A	Intronic alteration	WHT3040	1		
Intron 10	+42C→A	Intronic alteration	WHT3839	1		
Intron 12	−28T→C	Intronic alteration	WHT3058	1		
Intron 15	−64G→A	Intronic alteration	WHT3158	1		
Intron 21	−1G→A	Alteration of splicing acceptor site	WHT2445	1		
Intron 22	−37A→G	Intronic alteration	WHT3059	1		
Intron 24	+119G→A	Intronic alteration	WHT3158	1		
Intron 27	−55A→C	Intronic alteration	WHT2546	1		
Intron 28	−44A→G	Intronic alteration	WHT3864	1		
Exon 7	466A→G	Missense mutation, M152V				1
Intron 20	+16A→G	Intronic alteration				1
Intron 23	−44C→T	Intronic alteration			1	

AZ, azoospermic males.

a Number of individuals screened.

b +1 refers to the first base of a given intron, and −1, the last base.

We detected a significantly higher percentage of singleton variants in men with spermatogenic failure than in controls (7.3% versus 1.7%, *P *=* *0.007, Fisher’s exact test, Table1). Of the 21 singletons, 18 were found in azoospermic men and three in controls (Table1). The significantly higher prevalence of singleton variants in azoospermic men strongly suggests that *TEX11* is required for spermatogenesis in human.

With the exception of a frameshift mutation, 1258Ins(TT) in exon 16, and a splice site mutation, in intron 21, the mechanisms by which the singleton mutations may cause or predispose to azoospermia are not clear. Five of the singleton exonic mutations among azoospermic men were missense (W117R, V142I, Q172R, T244I, and V748A), and two were silent (Table1). The remaining 10 were intronic mutations with undetermined functional consequences except for the splice site mutation (−1G→A) at the consensus 3′ splice acceptor site in intron 21, which would be expected to abolish splicing (Table1).

### Meiotic arrest in an azoospermic man with a frameshift mutation in *TEX11*

**Figure 1:**
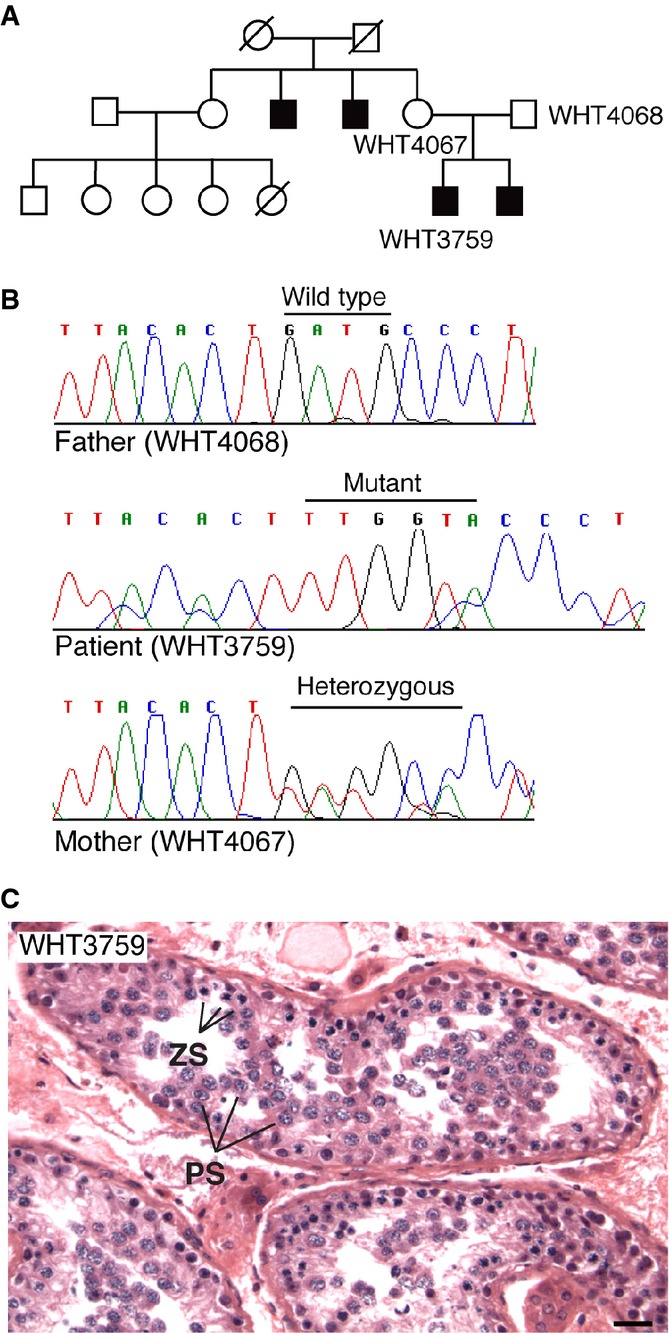
X-linked inheritance of a frameshift mutation in *TEX11* Family tree of the azoospermic male patient WHT3759. The patient’s brother was azoospermic with maturation arrest, and two maternal uncles were childless; DNA samples from these individuals could not be obtained. The patient’s paternal uncle and all five paternal aunts each had two or three children (not shown).

Among the singleton *TEX11* mutations identified in azoospermic men, a frameshift mutation in exon 16 would predictably impair TEX11 protein function, yielding a severely truncated protein comprising only the N-terminal half. The patient carrying this mutation (WHT3759) and his brother were azoospermic, and two maternal uncles were childless (Fig1A). Analysis of DNA samples from both parents of WHT3759 revealed that the mother was heterozygous for the mutation, whereas the father had the wild-type allele (Fig1B), demonstrating inheritance of the mutation from the mother. We could not obtain DNA samples from the patient’s brother or maternal uncles and thus were unable to determine whether these relatives carried the same mutation as the proband. Nevertheless, the data in this family are consistent with X-linked spermatogenic failure.

Complex frameshift mutation (patient WHT3759). The nucleotide sequence in exon 16 (in parentheses) changes from ACT(GATG)CCC to ACT(TTGGTA)CCC, resulting in a net insertion of two bases and generation of a *Kpn*I restriction site (underlined). We confirmed heterozygosity of the mother (WHT4067) by *Kpn*I digestion of PCR products (data not shown).

Testicular maturation arrest in the patient (WHT3759). Testicular tissue obtained by biopsy was sectioned and stained with hematoxylin/eosin. Seminiferous tubules contained meiotic germ cells such as zygotene (ZS, arrowheads) and pachytene spermatocytes (PS, arrows), but no post-meiotic germ cells such as round spermatids were detectable. Scale bar, 25 μm.

Histological analysis of a testis biopsy obtained from the azoospermic patient WHT3759 revealed meiotic arrest at the pachytene stage (Fig1C). No post-meiotic germ cells such as round spermatids and mature spermatozoa were observed in the seminiferous tubules, consistent with the diagnosis of azoospermia. Based on the histological data, we conclude that the primary defect caused by the *TEX11* frameshift mutation of this patient is meiotic arrest, corresponding to the phenotype observed in *Tex11*-null (*Tex11*^−/Y^) male mice (Yang *et al*, 2008).

### Experimental transfer of *Tex11* from the X chromosome to an autosome

**Figure 2:**
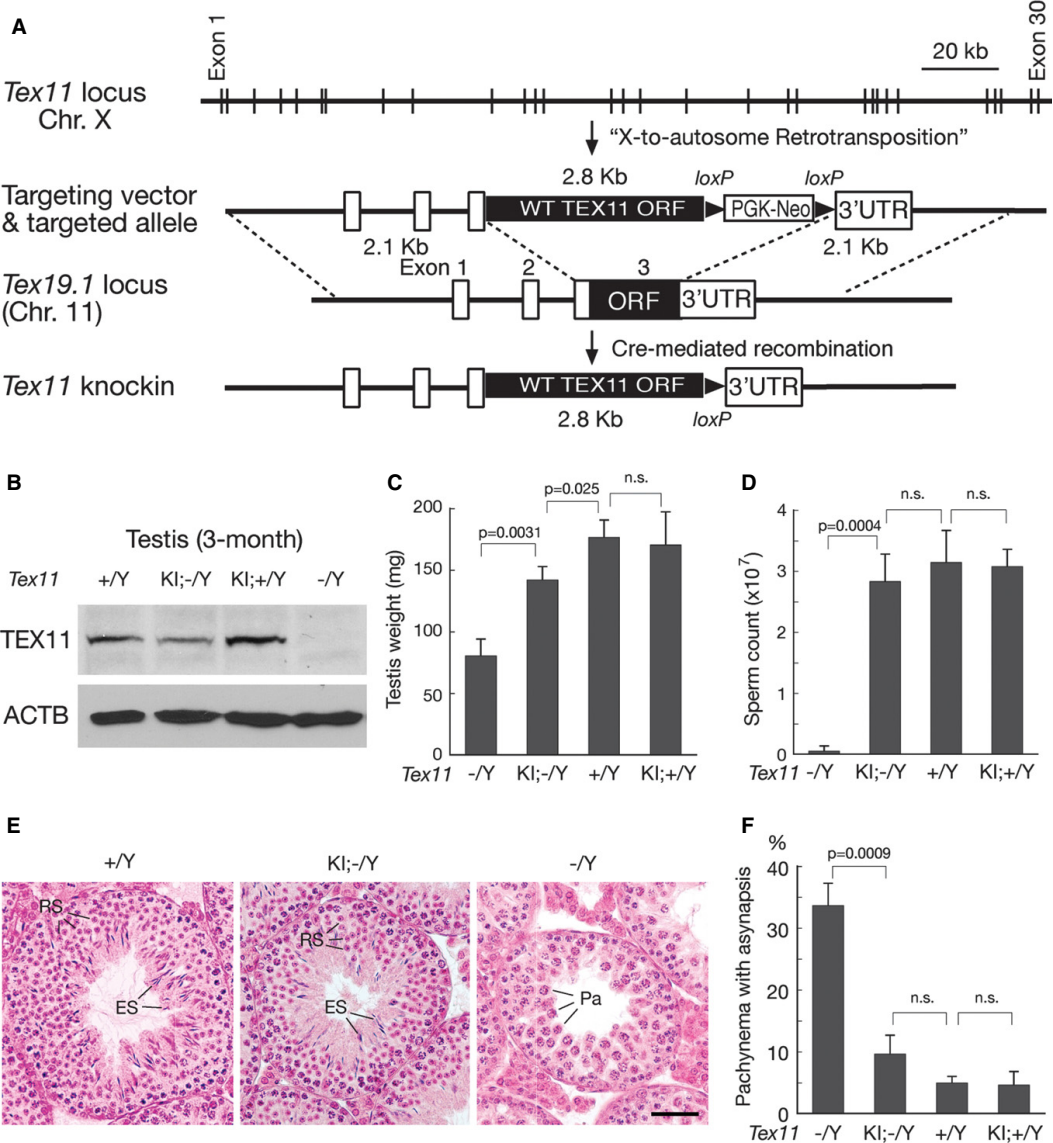
A wild-type *Tex11* knockin allele at the autosomal *Tex19.1* locus rescues male sterility in *Tex11*-null adult mice Schematic diagram of *Tex11* gene structure, *Tex19.1* gene structure, targeting vector and the final *Tex11* knockin allele containing the wild-type *Tex11* ORF.

To circumvent the inherent problems in the genetic dissection of human infertility as described earlier, we chose to analyze the consequences of mutant human *TEX11* alleles in genetically modified mice harboring analogous mutations in murine *Tex11*. The generation of mice with gene-specific mutations usually relies on gene targeting by homologous recombination in male (XY) embryonic stem (ES) cells and subsequent transmission of the modified allele through the germ line of male ES cell chimeras. However, this approach would predictably fail when modeling X-linked mutations causing male infertility, as these would not be transmitted through the male germ line. To overcome this impediment, we generated an experimental copy of the *Tex11* gene at an autosomal locus, placing a *Tex11* knockin allele under the transcriptional and translational control of *Tex19.1* (Chr. 11; Fig2A). We chose the *Tex19.1* locus for several reasons. First, *Tex19.1* and *Tex11* exhibit similar temporal expression patterns during spermatogenesis. Previous studies have shown that both genes are expressed in spermatogonia and early spermatocytes (Wang *et al*, 2005; Yang *et al*, 2008, 2010). Furthermore, the *Tex19.1* ORF is entirely encoded in one exon (exon 3) (Kuntz *et al*, 2008; Ollinger *et al*, 2008; Yang *et al*, 2010), permitting replacement of the *Tex19* ORF with the *Tex11* ORF (Fig2A). As *Tex19*^+/−^ mice display normal fertility (Ollinger *et al*, 2008; Yang *et al*, 2010), the deletion of one *Tex19.1* copy to generate the *Tex11* knockin allele would not be associated with phenotypic consequences.

Western blot analysis of TEX11 in the testes of 3-month-old mice with different *Tex11* gene dosages. ACTB serves as a loading control.

Testis weight of 3-month-old males.

Sperm count of 3-month-old males.

Histological analysis of testes from adult wild-type, *Tex11* KI/KO, and *Tex11*^−/Y^ males. RS, round spermatids; ES, elongating spermatids; Pa, pachytene spermatocytes. Scale bar, 50 μm.

The *Tex11* knockin allele rescues chromosomal synapsis defects in *Tex11*^−/Y^ spermatocytes in 3-month-old males. Chromosomal synapsis defects were assessed by SYCP1 and SYCP2 immunostaining of spread nuclei from 100 pachytene spermatocytes per male; for each genotype, three males were analyzed. Abbreviations for *Tex11* genotypes: −/Y, *Tex11* knockout (Yang *et al*, 2008); KI;−/Y, *Tex11* knockin and knockout; +/Y, wild type; KI;+/Y, *Tex11* knockin plus wild-type *Tex11*. Data information: All statistical analyses were performed using Student’s *t*-test. n.s.: not statistically significant.

### An autosomal *Tex11* knockin minigene rescues male infertility in adult *Tex11*^−/Y^ mice

We next determined whether the wild-type autosomal *Tex11* knockin minigene (*Tex19*^Tex11KI^) could functionally replace its X-linked progenitor and rescue infertility of azoospermic *Tex11*^−/Y^ males. In male meiotic germ cells, TEX11 localizes to recombination nodules and regulates both chromosomal synapsis and the formation of crossovers between homologous chromosomes. *Tex11*-deficient spermatocytes are eliminated at the pachytene and anaphase I stages of meiosis due to extensive asynapsis and a failure in chromosome segregation (Yang *et al*, 2008). We crossed *Tex19*^Tex11KI/+^ males (Fig2A) with *Tex11*^+/−^ females to generate *Tex19*^Tex11KI/+^ *Tex11*^−/Y^ males (hereafter referred to as *Tex11* KI/KO males). Western blot analysis confirmed that TEX11 protein was expressed in the testes of adult (3-month) KI/KO male mice albeit at lower levels compared to wild-type testes (Fig2B). *Tex11* knockin males (*Tex11* KI plus the wild-type *Tex1l* allele) exhibited higher testicular TEX11 protein levels compared to wild-type males. Therefore, TEX11 protein levels in these mouse models correlate with gene dosage (Fig2B). Adult males of the four genotypes (*Tex11*^−/Y^, *Tex11* KI/*Tex11*^−/Y^, *Tex11*^+/Y^, *Tex11* KI/*Tex11*^+/Y^) had comparable body weight (data not shown). Testes from adult KI/KO males weighed significantly more than those from *Tex11*-null males but less than those from wild-type males (Fig2C). Strikingly, the sperm count of 3-month-old KI/KO males was comparable to that of wild-type males (Fig2D). As expected, the KI/KO males were fertile. Histological analysis revealed that KI/KO and wild-type testes contained the full spectrum of spermatogenic cells including mature spermatozoa, whereas *Tex11*-null testes exhibited meiotic arrest of germ cells as previously reported (Fig2E) (Yang *et al*, 2008). Chromosomal synapsis defects caused by *Tex11* deficiency were rescued by the *Tex11* KI minigene (Fig2F). Taken together, we conclude that the autosomal *Tex11* KI minigene rescues meiotic arrest and male infertility in adult *Tex11*^−/Y^ mice.

### Defective meiosis in the first wave of spermatogenesis in *Tex11* KI/KO mice

**Figure 3:**
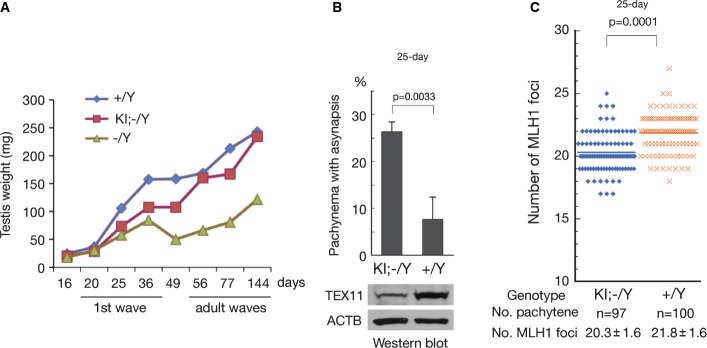
Defective meiotic progression in the first wave of spermatogenesis in *Tex11* KI/KO males The knockin allele expresses the wild-type *Tex11* ORF.
Reduced testis weight in juvenile but not adult *Tex11* KI; −/Y males. The time frames of the first wave and adult waves of spermatogenesis are indicated.

Testes from 8-week-old *Tex11* KI/KO males were comparable in size and weight to wild-type testes (Fig3A). However, testes from juvenile *Tex11* KI/KO males (postnatal day 25, 36, or 49) were much smaller than wild-type testes (Fig3A), suggesting a developmental delay or a failure in the first wave of spermatogenesis in *Tex11* KI/KO males. Spermatogenesis proceeds through distinct stages that include mitotic proliferation of spermatogonial stem cells, meiotic division of spermatocytes, and spermiogenesis of haploid spermatids; this occurs in a locally synchronized and coordinated manner described as spermatogenic waves (McCarrey, 1993). In juvenile mice, the first wave of spermatogenesis is initiated within 1 week after birth, producing mature spermatozoa by postnatal day 35. This wave bypasses a self-renewing spermatogonia stage and displays a synchronized appearance of differentiating germ cells in seminiferous tubules (de Rooij, 1998). In adult mice, waves of spermatogenesis are not synchronized among different seminiferous tubules. In *Tex11*-null male mice, lack of TEX11 affects juvenile and adult spermatogenic waves equally (Yang *et al*, 2008). In mature *Tex11* KI/KO male mice, the chromosomal asynapsis defects caused by TEX11 deficiency were rescued by the *Tex11* KI allele (Fig2F). However, analysis of spermatocytes from juvenile *Tex11* KI/KO males revealed that chromosomal synapsis remained severely impaired at day 25, that is, during the first spermatogenic wave (Fig3B and Supplementary Fig S2). Evaluation of the number of MLH1 foci, which mark the site of future meiotic crossovers (Anderson *et al*, 1999), indicated that the meiotic recombination defect in juvenile testis was largely but not fully rescued by the *Tex11* KI minigene (Fig3C). These results suggest that defective meiosis rather than developmental delay causes the severe impairment of the first spermatogenic wave in juvenile *Tex11* KI/KO males. Western blot analysis revealed substantially lower TEX11 protein levels in juvenile (day 25) KI/KO testes compared to wild-type testes (Fig3B). These results suggest that the amount of TEX11 protein expressed from the *Tex11* KI allele was too low to fully rescue meiotic defects in *Tex11*-null juveniles (Fig3B), but was expressed at a higher level in adult mice, which is sufficient for meiotic progression in adult *Tex11* KI/KO males (Fig2B). Alternatively, different threshold levels of TEX11 may be needed for progression of meiosis during the first wave of spermatogenesis compared to adult waves of spermatogenesis.

Increased chromosomal asynapsis in spermatocytes and reduced levels of TEX11 protein in the testes of 25-day-old *Tex11* KI/KO males. Chromosomal synapsis defects were assessed by SYCP1 and SYCP2 immunostaining of spread nuclei from 100 pachytene spermatocytes per male; for each genotype, three males were analyzed.

Pachytene spermatocytes from 25-day-old *Tex11* KI/KO males contain significantly fewer MLH1 foci compared to wild-type spermatocytes. Values are shown as average ± standard deviation. Error bar is standard deviation.

Data information: Statistical analyses were performed with Student’s *t*-test.

### Meiotic recombination levels are sensitive to the gene dosage of *Tex11*

**Figure 4:**
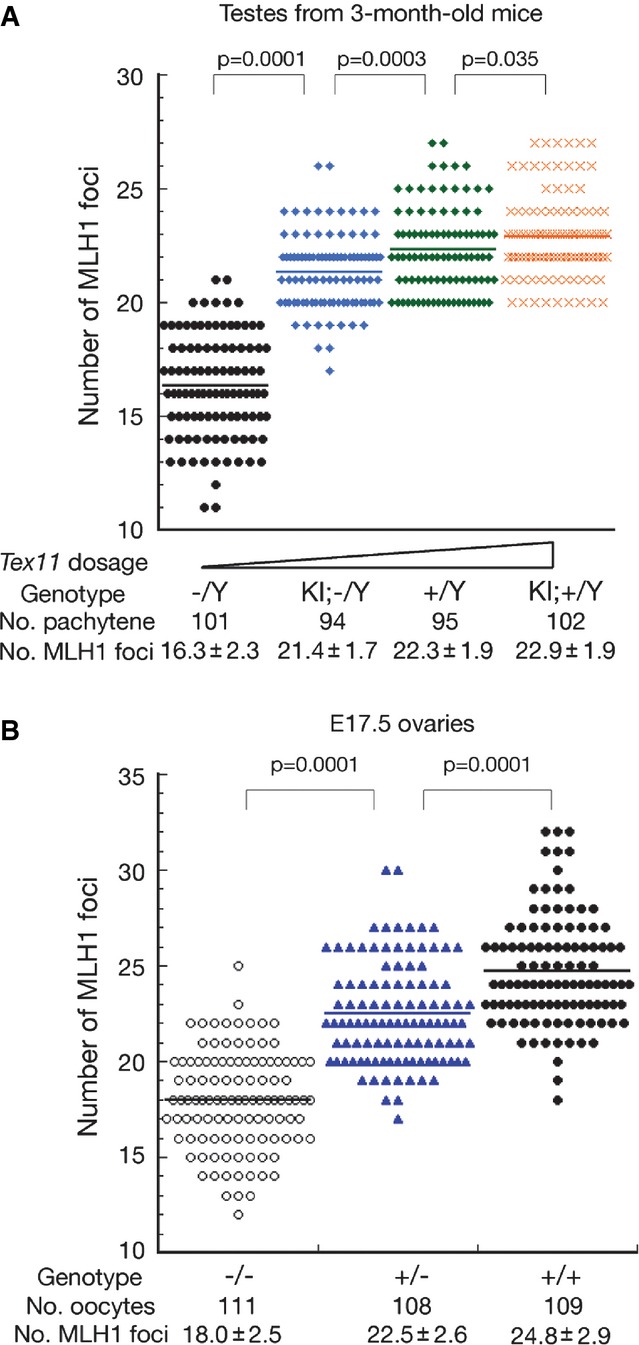
*Tex11* dosage affects the number of MLH1 foci in meiotic germ cells in both sexes The number of MLH1 foci in pachytene spermatocytes from 3-month-old males positively correlates with increasing *Tex11* gene dosage.

The presence of fewer MLH1 foci in spermatocytes from *Tex11* KI/KO juvenile mice compared to wild type suggested that *Tex11* dosage might influence the rate of meiotic recombination (Fig3C). To further test this hypothesis, we analyzed the recombination rate in spermatocytes from 3-month-old males with increasing *Tex11* gene dosages (genotypes: *Tex11*^−/Y^, *Tex11* KI/*Tex11*^−/Y^, *Tex11*^+/Y^, *Tex11* KI/*Tex11*^+/Y^; Fig4A and Supplementary Fig S3). The recombination rate was significantly different among these males, with the lowest rate in *Tex11*^−/Y^ males and the highest rate in *Tex11* KI/*Tex11*^+/Y^ males, revealing a positive correlation between meiotic recombination rate and *Tex11* dosage in males.

Quantification of MLH1 foci in pachytene oocytes from the ovaries of *Tex11*^−/−^, *Tex11*^+/−^, and wild-type embryonic day-17.5 (E17.5) fetuses reveals a positive correlation of *Tex11* gene dosage and the number of MLH1 foci. Data information: Statistical analyses were performed with Student’s *t*-test.

In female germ cells, both X chromosomes are active such that both copies of the *Tex11* gene can be transcribed. *Tex11* deficiency reduces the number of MLH1 foci in fetal oocytes (Yang *et al*, 2008). To ascertain whether the meiotic recombination rate in females depends on *Tex11* gene dosage, we evaluated recombination frequencies in *Tex11*^−/−^, *Tex11*^+/−^, and wild-type oocytes (Fig4B). The average number of MLH1 foci in *Tex11*^+/−^ oocytes was significantly higher than in *Tex11*^−/−^ oocytes but significantly lower than in wild-type oocytes, demonstrating that meiotic recombination rate in females is strongly affected by the *Tex11* gene dosage. In conclusion, meiotic recombination rates in both sexes are sensitive to the *Tex11* gene dosage.

### A *TEX11* missense mutation found in an infertile man causes defects in chromosomal synapsis

**Figure 5:**
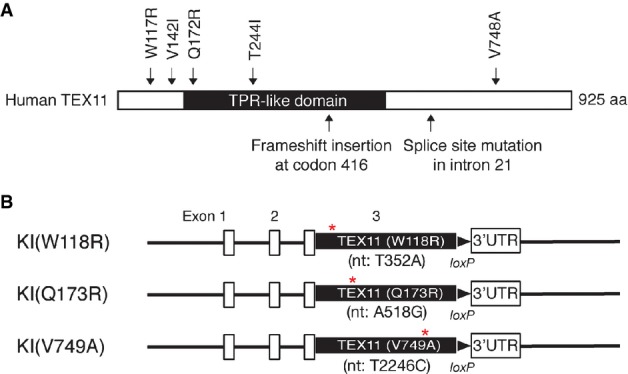
Modeling human male infertility in mice using the autosomal knockin approach Schematic representation of missense and nonsense mutations in human *TEX11* found exclusively in men with azoospermia. The full-length TEX11 protein (GenBank accession number: NP_112566) contains 925 residues. The tetratricopeptide-like (TPR-like) helical domain, found in proteins that form large complexes, extends from residues 161 through 499 (Blatch & Lassle, 1999). Four of the residues mutated in azoospermic men (W117, V142, Q172, and V748) are conserved between human and mouse TEX11 proteins (Supplementary Fig S1).

We identified five *TEX11* missense mutations (W117R, V142I, Q172R, T244I, V748A) in azoospermic men (Table1 and Fig5A) that were not detected in any of the 175 control men, implying that they are likely to be genetic causes of infertility in humans. By sequence alignment analysis of TEX11 homologues from diverse species (human, mouse, rat, horse, cat, dog, marsupial, chicken, and fish), we found that three of these mutations affect residues that are highly evolutionary conserved and may therefore be important for functional and/or structural integrity (W117, Q172, and V748; Supplementary Fig S1). We therefore evaluated the consequences of missense mutations at these residues (W117R, Q172R, and V748A) *in vivo* by generating knockin mice harboring mutant *Tex11* “retrogenes” at the *Tex19.1* locus.

Generation of three lines of *Tex11* knockin mice. Each harbors a single amino acid substitution in TEX11 (indicated by asterisks) analogous to a missense mutation identified in a human patient. Nucleotide and respective amino acid changes are indicated. Exons 1, 2, and 3′UTR are from the mouse *Tex19.1* gene.

Using the same experimental approach as for the wild-type *Tex11* KI allele (Fig2A), we generated three different KI mouse lines, each harboring one TEX11 point mutation (W118R, Q173R, or V749A) (Fig5B) analogous to the human mutations. All three KI alleles were targeted to the *Tex19* locus through homologous recombination in ES cells and thus were under the transcriptional and translational control of the *Tex19* locus (Fig5B). These three alleles are referred to as KI(W118R), KI(Q173R), and KI(V749A), respectively. Mutant KI males (*Tex19*^Tex11KI(mutant)/+^ *Tex11*^+/Y^) were fertile, suggesting that none of these point mutations are dominant negative. We then crossed KI males with *Tex11*^+/−^ females to generate KI/KO males.

**Figure 6:**
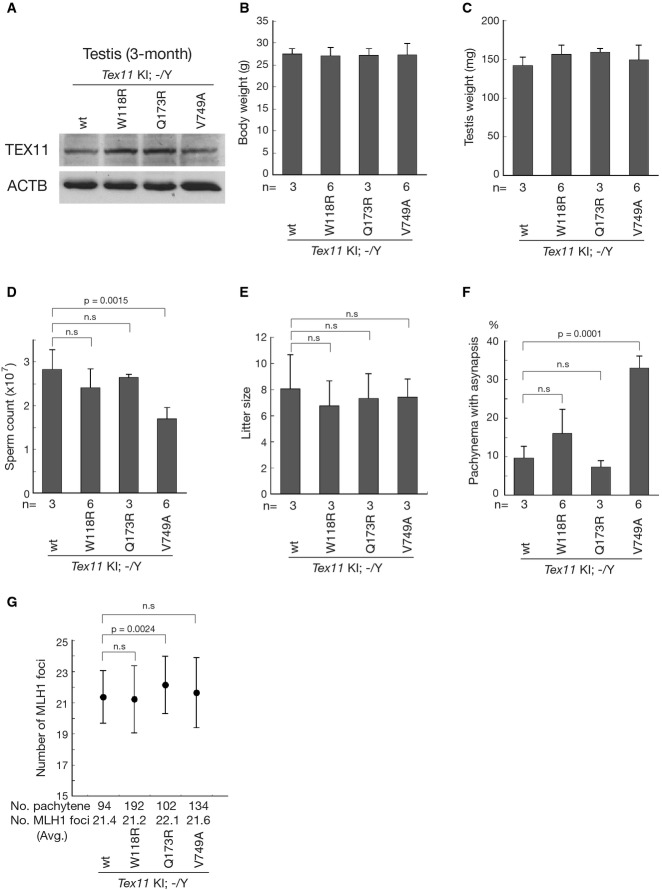
A single amino acid change (V749A) in TEX11 causes severe defects in chromosomal synapsis in mice Data shown are from 3-month-old *Tex11* KI/KO males with wild-type or mutant knockin allele (*n*, number of males analyzed per genotype) except for mating test (E).
A Western blot analysis reveals comparable levels of TEX11 protein in the testes of wild-type and point mutant KI/KO males. ACTB serves as a control.

Western blot analysis of testes from mature (3-month-old) males confirmed comparable expression levels of TEX11 from all KI alleles (Fig6A). Mature KI/KO males with the three different *Tex11* KI alleles (three point mutations) were comparable to KI/KO males with a wild-type *Tex11* KI allele (hereafter referred to as wild-type KI/KO) in respect to body weight, testis weight, and fecundity (Fig6B,C and E). Sperm counts of KI(W118R)/KO and KI(Q173R)/KO males were also similar to wild-type KI/KO males (Fig6D), correlating with rescue of chromosomal synapsis defects in males of these genotypes (Fig6F and Supplementary Fig S4). Meiotic recombination rates, reflected by the number of MLH foci in pachytene stage spermatocytes, were comparable between KI(W118R)/KO and wild-type KI/KO males. Intriguingly, pachytene cells from KI(Q173R)/KO males contained more MLH foci than wild-type KI/KO germ cells, indicating a higher rate of recombination (Fig6G). These results suggest that the *TEX11* mutations (W117R and Q172R) may not cause male infertility in humans.

B–D Body weight (B), testis weight (C), and significantly reduced sperm count (per pair of epididymides) in *Tex11* KI(V749A)/KO males (D).

E Mating test of 3- to 5-month-old males.

F Dramatically increased chromosomal asynapsis in spermatocytes from *Tex11* KI(V749A)/KO males. 100 pachytene spermatocytes per mouse were examined by surface spread analysis.

G Number of MLH1 foci in pachytene spermatocytes. Note a significantly higher number of MLH1 foci in spermatocytes from KI(Q173R)/KO males. Data information: Values are shown as average ± standard deviation. n.s.: not statistically significant.

In striking contrast, the sperm count of KI(V749A)/KO males was significantly reduced compared to controls (Fig6D). Surface spread analysis of pachytene spermatocytes revealed a similar proportion of asynapsis in germ cells from KI(V749A)/KO (33%; Fig6F and Supplementary Fig S4) and KO males (34%; Fig2F), revealing that the KI(V749A)/KO mutant phenocopies the KO mutant in terms of chromosomal synapsis in mice (Yang *et al*, 2008). Consistent with these observations, histological analysis of a testis biopsy obtained from patient WHT2499 carrying the *TEX11* V748A missense mutation (Table1) revealed meiotic arrest at the pachytene stage (Supplementary Fig S5). However, the meiotic recombination rate in KI(V749A)/KO males was comparable to the control (Fig6G). We previously showed that TEX11 regulates two distinct processes during meiosis: chromosomal synapsis and meiotic recombination (Yang *et al*, 2008). Our current data therefore identify the single amino acid change (V749A) in TEX11 as a separation-of-function mutation that disrupts chromosomal synapsis but not meiotic recombination. Significantly, our results strongly support that the human TEX11 V748A mutation is likely a genetic cause of infertility in azoospermic men.

## Discussion

Our studies show that *TEX11*, an X-linked meiosis-specific gene, is mutated in azoospermic men. A conservative calculation that considers only three *TEX11* mutations (frameshift mutation in exon 16, splice site mutation in intron 21, and V748A missense mutation) indicates an infertility-causing mutation frequency in human *TEX11* of ∼1% (three mutations/246 azoospermic men screened, Table1). Given that hundreds, if not thousands, of genes specifically regulate fertility, finding a causative mutation frequency of 1% in *TEX11* is highly significant. This frequency is comparable with the mutation frequency of BRCA1 (∼2%) in breast cancers (Kurian, 2010). Finding a high mutation frequency of *TEX11* among infertile men is not entirely unexpected: *TEX11* is X-linked, such that any inherited or *de novo* mutations that impair the function of this essential fertility factor would manifest as infertility.

Histological analysis of testis biopsies from two azoospermic men with *TEX11* mutations revealed meiotic arrest at the pachytene stage, indicating that TEX11 plays a critical role in human meiosis (Fig1C and Supplementary Fig S5). We have previously shown that mouse TEX11 protein localizes to foci on meiotic chromosomes and that TEX11 promotes homologous recombination and chromosomal synapsis (Yang *et al*, 2008). Mutations in *SPO22/ZIP4*, which are the budding yeast and *Arabidopsis* homologues of *TEX11,* lead to defects in meiosis (Tsubouchi *et al*, 2006; Chelysheva *et al*, 2007). TEX11 therefore plays an evolutionarily conserved role in meiosis from budding yeast to humans. It is noteworthy that the azoospermic man with the V748A mutation exhibits complete meiotic arrest (Supplementary Fig S5), whereas *Tex11* KI(V749A)/KO mice displayed severe meiotic defects but no complete meiotic arrest. The differential effect of this missense mutation on the fertility of mouse and human may be attributed to the species-specific requirement or context, as *Tex11* is a rapidly evolving gene with only 56% protein sequence identity between mouse and human.

Crossovers are formed through at least two pathways: MLH1-dependent and Mus81-dependent (de los Santos *et al*, 2003; Holloway *et al*, 2008). The majority of crossovers in mice is processed through the MLH1-dependent pathway and is subject to crossover interference, a phenomenon that ensures wide spacing of crossovers on the same chromosome (Holliday, 1977; Bishop & Zickler, 2004). ZIP4/TEX11 belongs to the ZMM protein group and thus promotes crossover via the MLH1-dependent pathway (Perry *et al*, 2005; Tsubouchi *et al*, 2006; Chelysheva *et al*, 2007). In fact, disruption of ZIP4/TEX11 in yeast, *Arabidopsis*, and mouse causes reduction in MLH1-dependent crossovers (Tsubouchi *et al*, 2006; Chelysheva *et al*, 2007; Yang *et al*, 2008). Notably, the effect of *Tex11* gene dosage on the number of MLH1 foci in mouse is nonlinear. This nonlinear effect may be largely attributed to crossover homeostasis (Martini *et al*, 2006; Zhang *et al*, 2014). Although Mus81-dependent crossovers are not affected in *Arabidopsis Zip4* mutant (Chelysheva *et al*, 2007), further studies are needed to examine the formation of Mus81-dependent crossovers in *Tex11*-deficient and knockin mutant mice.

Recent linkage-based studies in humans and mice suggest that multiple loci regulate the levels of genome-wide meiotic recombination (Kong *et al*, 2008; Chowdhury *et al*, 2009; Murdoch *et al*, 2010). Variants in RNF212, a SUMO E3 ligase, is associated with recombination rate in human populations (Kong *et al*, 2008). Mouse RNF212 localizes to a subset of recombination sites (Lake & Hawley, 2013; Reynolds *et al*, 2013). HEI10, a ubiquitin E3 ligase, regulates meiotic recombination in mouse, rice, and *Arabidopsis* (Ward *et al*, 2007; Chelysheva *et al*, 2012; Wang *et al*, 2012). Interestingly, the levels of crossovers are sensitive to the gene dosage of *Rnf212* and *Hei10* (Lake & Hawley, 2013; Reynolds *et al*, 2013; Qiao *et al*, 2014). Seven recombination-associated loci have been mapped in the mouse genome, and the genetic locus with the highest LOD score in mouse maps close to *Tex11* on the X chromosome (Murdoch *et al*, 2010). Non-synonymous *Tex11* SNPs are associated with testicular size in cattle (Lyons *et al*, 2014). Our current results demonstrate that recombination rates in both sexes are sensitive to TEX11 levels. In addition, a missense mutation (Q173R) in *Tex11* increases the recombination rate in mice (Fig6G). Therefore, different expression levels of *TEX11* or single amino acid substitutions may contribute to the variable genome-wide meiotic recombination rates between sexes and among individuals, and as such, low-expressing *TEX11* alleles could be genetic causes of male infertility in humans.

We find that the engineered *Tex11* minigene on the autosome rescues the infertility caused by the X-linked *Tex11* deletion. The significance of this finding is two-fold. First, genetic modification of this allele can be used as a strategy to determine the consequences of human *TEX11* mutations *in vivo* by introducing analogous mutations into the autosomal *Tex11* knockin allele. Secondly, this allele resembles and therefore models endogenous testis-specific X-to autosomal retrogenes. These genes originate from the retrotransposition of X-linked genes to autosomes during evolution and exhibit testis-specific expression patterns. A common hypothesis for the evolution of X-to autosomal retrogenes is that they provide a backup source for gene expression during mammalian meiosis, which involves silencing of the X chromosome in males (McCarrey & Thomas, 1987; Emerson *et al*, 2004; Wang, 2004; Turner, 2007). The autosomal *Tex11* knockin allele generated in our study carries the hallmarks of an X-to autosomal retrogene, that is, lack of its introns, and therefore provides the first direct genetic evidence that such a gene can substitute for the function of its X-linked “ancestral” gene.

## Materials and Methods

### Ethical considerations and patient consents

The human mutation screening protocol was approved by the Institutional Review Board of the Massachusetts Institute of Technology. Informed consent was obtained from all participants. Experiments conformed to the principles set out in the WMA Declaration of Helsinki and the Department of Health and Human Services Belmont Report. Standard barrier mouse housing conditions and all experiments involving mice were approved by the Institutional Animal Care and Use Committee of the University of Pennsylvania. Animals were used based on genotypes. No randomization and no blinding were used.

### Population samples

We studied 246 patients with non-obstructive azoospermia. We excluded patients known to have had any of the following conditions or treatments that cause or predispose to spermatogenic failure: Y-chromosomal deletions (Reijo *et al*, 1995, 1996; Vogt *et al*, 1996; Kuroda-Kawaguchi *et al*, 2001; Repping *et al*, 2002); a 47, XXY karyotype; orchitis; cryptorchidism; radiotherapy; or chemotherapy. The 175 control subjects included men known to have fathered children (*n* = 93) and men of unknown fertility selected to represent worldwide genetic diversity based on their Y-chromosomal haplotypes (*n* = 82, samples from the NIH polymorphism discovery panel, Coriell Cell Repositories, and from our collection; Collins *et al*, 1998). We prepared DNA from peripheral blood leukocytes or EBV-transformed lymphoblastoid cell lines.

### Mutation screening

The *TEX11* exon/intron structure was determined by alignment of the *TEX11* cDNA sequence (NM_031276) with its genomic sequence (Wang *et al*, 2001). We amplified the *TEX11* coding exons (exons 2 through 30) by PCR using 28 primer pairs (GenBank dbSTS accession numbers: BV703476–BV703503). PCR was performed in a 25 μl reaction with 12.5 ng genomic DNA (94°C, 30 s; 56°C, 30 s; 72°C, 90 s; 35 cycles). PCR products were purified by Sephadex S-300 gel filtration. 12.5 μl of purified PCR product was sequenced in a 25 μl reaction using one of the PCR primers and ABI BigDye according to the manufacturer’s instructions. Reaction products were separated and read on an ABI 3700 sequencer. Sequence analysis was performed using the Sequencher software (Gene Codes Corporation), and sequence variants were identified by manual inspection of aligned sequences.

### Generation of *Tex11* knockin mice

The two homologous arms (2.1 kb each) of the *Tex11* knockin targeting construct were amplified from a *Tex19.1*-positive BAC clone (RP23-400P17) by high-fidelity PCR and were subcloned into the NeoA plasmid to flank a floxed PGK-Neo selection cassette, resulting in the parent vector pUP104-12. The mouse *Tex11* ORF was subcloned into pUP104/*Cal*I-*EcoR*V upstream of the PGK-Neo cassette, resulting in pUP115 (Fig2). The construct was verified by sequencing. Hybrid V6.5 XY ES cells (C57BL/6 × 129/sv) were electroporated with the linearized *Tex11* knockin targeting construct (pUP115/*Not*I), followed by culture in the presence of G418 (350 μg/ml). Seven days after electroporation, 96 G418-resistant ES cell clones were picked and screened by PCR for homologous recombination on both sides, identifying seven homologously targeted ES cell clones. Two targeted ES cell clones (C4 and E8) were injected into B6C3F1 (Taconic) blastocysts that were subsequently transferred to the uteri of pseudopregnant ICR females. Male chimeras were bred with *Actb*-Cre females to delete the PGK-Neo cassette, and germ-line transmission of the wild-type *Tex11* knockin allele was obtained from chimeras derived from both ES cell clones.

To generate *Tex11* alleles with missense mutations, nucleotide changes were introduced into the *Tex11* ORF by overlapping PCR, followed by subcloning of the mutant *Tex11* ORF into pUP104-12. The final *Tex11* knockin targeting constructs for mutations T352A, A518G, and T2246C were pUP106-1, pUP107-4, and pUP108-5. Sequencing of the final constructs confirmed each desired mutation and revealed no other mutations. ES cells were targeted and screened as described above. One ES cell clone for each point mutation was injected into blastocysts. ES cell clones 1C2, 2D4, and 3G2 contained the *Tex11* knockin allele bearing mutations T352A, A518G, and T2246C, respectively. These three nucleotide mutations correspond to amino acid changes W118R, Q173R, and V749A, respectively (Fig5B). All knockin alleles were transmitted through the germ line from male chimeras, and point mutations were further confirmed by DNA sequencing of amplicons from tail genomic DNA. All knockin mice used in the study were from colonies that had been backcrossed to C57BL/6J for three generations. Offspring were genotyped by PCR of tail genomic DNA with the following primers: *Tex11* knockin allele with PGK-Neo (510 bp), GCACCCTCAAAACAAGCTATG and CCTACCGGTGGATGTGGAATGTGTG; *Tex11* knockin allele without PGK-Neo (252 bp), GCACCCTCAAAACAAGCTATG and CTGAGCTTTAGTGTCTCAGG; *Tex11* knockout allele (530 bp), ACTGTGTTACACTAGGTTGGA and TGAGGTCTGAAATCTGAGTTG.

### Histological and nuclear surface spread analysis

For histology, testes were fixed in Bouin’s solution, embedded in paraffin, sectioned, processed, and stained with hematoxylin and eosin. For meiotic nuclear surface spread analysis, spermatocytes or prophase I oocytes were prepared using the dry-down method as previously described (Peters *et al*, 1997; Kolas *et al*, 2005). Spread nuclei were immunostained with the following primary antibodies: Anti-SYCP1 (1:50; catalog no. ab15090, Abcam), anti-SYCP2 (1:100, sera 1918 and GP21; Yang *et al*, 2006), anti-MLH1 (1:50; catalog no. 550838, clone G168-15; BD Biosciences), followed by detection with FITC- and Texas red-conjugated secondary antibodies. Images were captured on a digital camera coupled to a Zeiss Axioskop 40 fluorescence microscope. The number of MLH1 foci per nucleus was counted.

### Mating tests and sperm count

Each adult male (three males per genotype; Fig6E) at the age of 3–5 months was housed with two 8-week-old wild-type C57BL/6J females for 4 months. Mice were checked daily, and litter size was recorded. Sperm count was performed as previously described (Cheng *et al*, 2007).

### Western blot analysis

Testicular protein extracts were prepared by homogenization of testes from 3-month- or 25-day-old mice in SDS–PAGE buffer. Testicular protein (30 μg) was separated on a SDS–PAGE gel and blotted onto a nitrocellulose membrane. The blot was probed with the following primary antibodies: anti-TEX11 (Yang *et al*, 2008) and anti-ACTB (1 : 7,500; catalog no. A5441, clone AC-15; Sigma).

### Statistics

All data were analyzed using GraphPad Prism (GraphPad Software Inc) and KaleidaGraph (Synergy Software). Statistical analysis of singleton variants in infertile and control men was conducted using Fisher’s exact test (Table1). Student’s *t*-test was performed for body weight, testis weight, sperm count, mating test, chromosomal asynapsis, and the number of MLH1 foci. Normal distribution of the number of MLH1 foci was tested using Shapiro–Wilk test and D’Agostino’s *K*-squared test in GraphPad Prism. A *P*-value ≤ 0.05 was considered significant.

**The paper explained**

**Problem**

Infertility is a worldwide reproductive health issue. Azoospermia, characterized by the absence of sperm in semen, is a severe form of male infertility. The cause for the majority of azoospermia is unknown and likely to be genetic. Genetic studies of azoospermia in humans is complicated by the presence of hundreds, if not thousands, of candidate genes.

**Results**

We sequenced azoospermic patients (with no known causes) for mutations in *TEX11*, an X chromosome-linked germ cell-specific gene. We found one frameshift mutation, one point mutation at a splicing acceptor site, and a number of missense mutations in infertile patients. The frameshift mutation in the reported patient was passed from the mother, consistent with X-linked inheritance. Further analysis using mouse as an *in vivo* model demonstrated that one missense mutation (V748A) impairs meiosis. Our results show that mutations in the *TEX11* gene account for 1% of infertility in non-obstructive azoospermic men. Furthermore, genome-wide recombination rates in both sexes depend on levels of TEX11 protein.

**Impact**

Our results suggest that mutations in TEX11 underlie non-obstructive azoospermia in a significant fraction of men. Identification of genetic causes of male infertility would improve genetic counseling for patients seeking infertility treatment.

## For more information

Online Mendelian Inheritance in Man (OMIM)/TEX11, http://omim.org/entry/300311

GenBank SNP database, http://www.ncbi.nlm.nih.gov/projects/SNP/

Coriell Cell Repositories, https://catalog.coriell.org/

## Supporting Information

**Supplementary Information**

**Review Process File**
