# Supplementary material for: TEX11 is mutated in infertile men with azoospermia and regulates genome-wide recombination rates in mouse
Source: EMBO Mol Med. 2015 Jul 1;7(9):1198–210. doi: 10.15252/emmm.201404967 (PMC4568952; doi:10.15252/emmm.201404967)

Supplementary Information

Table of Contents

Supplementary Figure Legends 2

Supplementary Table 3

Supplementary Figures 4

Supplementary Figure Legends

Supplementary Figure 1

Alignment of TEX11 protein sequences. Multiple sequence alignment was performed using CLUSTAL 2.0.9. Red font marks residues affected by the five *TEX11* missense mutations identified in infertile men.

Supplementary Figure 2

Analysis of chromosomal synapsis in spermatocytes from 25-day-old wild type and *Tex11* KI(WT);-Y mice. Surface spread nuclei from spermatocytes were immunostained with anti-SYCP1 and anti-SYCP2 antibodies. Representative images of spermatocytes at the pachytene stage are shown. XY chromosomes are indicated. This supplementary figure is related to Figure 3B.

Supplementary Figure 3

Number of MLH1 foci in spermatocytes from 3-month-old males. Representative images are shown for the following *Tex11* genotypes: A) -/Y, 17 MLH1 foci; B) KI;-/Y, 21 MLH1 foci; C) +/Y, 22 MLH1 foci; D) KI;+/Y, 26 MLH1 foci. This supplementary figure is related to Figure 4A.

Supplementary Figure 4

Analysis of chromosomal synapsis in spermatocytes from 3-month-old male mice. Surface spread nuclei from spermatocytes were immunostained with anti-SYCP1 and anti-SYCP2 antibodies. XY chromosomes are indicated (A-C). Representative images are shown for the following *Tex11* KI/KO (KI;-/Y) genotypes: A) wt; B) W118R; C) Q173R; D) KIV749A. The spermatocyte (D) displayed extensive chromosomal asynapsis. This supplementary figure is related to Figure 6E.

Supplementary Figure 5

Testicular maturation arrest in an infertile male patient with V748A mutation in *TEX11* (WHT2499). Testicular tissue was obtained by biopsy, sectioned, and stained with hematoxylin/eosin. The biopsy sample shows absence of post-meiotic germ cells such as round spermatids in the seminiferous tubule, whereas meiotic stage spermatocytes such as at the pachytene stage (arrows) are present. The boxed tubule in panel A is enlarged in panel B. Scale bar, 50 μm.

Supplementary Table 1 Common sequence variants in human *TEX11* gene.

Position	Nucleotide change	Resultant change	Infertile males AZ 246*	Normal males Fertile 93*	Normal males NIH diversity 82*
Exon 6	344A→G	Missense mutation, K115R; SNP: rs6525433	14[#]	5	20
Exon 16	1306G→A	Missense mutation, E436K; SNP: rs4844247	11	4	10
Exon 26	2274A→T	Silent mutation	1		1
Exon 28	2496T→G	Missense mutation, D832E			2
Exon 28	2496T→C	Silent mutation; SNP: rs16991177	13	4	9
Exon 30	123T→C 3'UTR	Alteration in the first AATAAA signal	2	1	0
Intron 4	+35G→A	Intronic alteration; SNP: rs5937008	118	34	29
Intron 4	-22T→C	Intronic alteration	7	1	9
Intron 4	-24C→T	Intronic alteration	1	1	3
Intron 7	-26T→C	Intronic alteration; SNP: rs17301944	25	9	2
Intron 8	+11T→A	Intronic alteration	1	1	1
Intron 11	-27T→C	Intronic SNP: rs6653304	12	5	2
Intron 16	+48A→T	Intronic SNP: rs5980996	38	23	13
Intron 18	-25A→G	Intronic SNP: rs1325094	40	24	14
Intron 18	-23T→C	Intronic SNP: rs1325094	88	39	21
Intron 21	-10T→A**	Intronic alteration	2		
Intron 24	+89T→C	Intronic alteration			2
Intron 28	+58T→C	Intronic SNP	8	3	12
Intron 28	-3, -4 TC→AT	Intronic SNP: rs1536250, rs1536251	15	4	2

Notes: *Number of individuals screened; AZ, azoospermic males. **+1 refers to the first base of a given intron, and –1 the last base.

[#]Number of males with the given variant.

Supplementary Figure 1 Alignment of TEX11 protein sequences

Species		Accession No.
Human		NM_031276
Mouse		NM_031384
Macaq	Macaque	AB074448
Dog		XM_844423
Horse		XM_001490344
Cow		XM_593293
Rat		XM_576992
Oposs	Monodelphis domestica	XP_001368341
Chick	Gallus gallus	XM_420206
Danio	Danio rerio	XM_001922537
Tetra	Tetraodon nigroviridis	CAG09799
Branch	Branchiostoma floridae	XM_002210899
Coral	Acropora millepora	EZ003728
Nemat	Nematostella vectensis	XM_001630824

Multiple sequence alignment was performed using CLUSTAL 2.0.9.

```
Human     ------------------------------------MDNDDFFSMDFKEVVENLVTNDNS  24
Mouse     -----------------------------------MDRITDFYFLDFRESVKTLIITGNS  25
Macaq     ------------------------------------------------------------  1
Dog       ----------------MTSLWEEVPSARRPALWIEFVERRLWLSWSCGEIVENLIVKDNS  44
Horse     -----------------------------------------------MKIIENLIVKGNS  13
Cow       ---------------------------------------MSLLSFVISETIENLIATDIS  21
Rat       -----------------------------------------------------MIITGNS  7
Oposs     ------------------------------------------------------------  1
Chick     ------------------------------------------------------------  1
Danio     ---------------------------------------MEQFAVEVKDLSEKLLHRQSH  21
Tetra     ------------------------------------------------------------  1
Branch    MHVFLLVFSTDLLRLLFKFNFKNLYLFFCRLKALEKETLPKCQATMIKALEKDKDEDKVA  60
Coral     ------------------------------------------------------------  1
Nemat     ------------------------------------------------------------  1
```

```
Human    PNIPEAIDRLFSDIANINRESMAEITDIQIEEMAVNLWNWALTIGGGWLVNEEQKIRLHY  84
Mouse    WRLQEMIDRFFTNISNFNRESLTEIQNIQIEEIAVNLWNWAVTKRVELSVRKNQAAKLCY  85
Macaq    --------MIFFPWT------------LKIEEMAVNLWNWALTIGGGWHVNEERKIKLHY  40
Dog      PSIPEAINRLFTDIANINRESIAEIQDAQVEEMAVNLWNWAVTKRVDLVINEEQKAKLWY  104
Horse    PSIHEAIDRLFMDIANINRESMAEMQDAQVEEMAVNLWNWAVTRRVGLVISEEQKAKLQH  73
Cow      PTTPAAIDRLFMDIGNINRESMAEIQDSQIEEMAVNLWNWIITNNIGLVINEEQKAKVRH  81
Rat      WRLHEMIDKFFMNISNFNRKSLTEVQNIQIEEIAVNLWNWATAKRLELSVRKSQAAKLCY  67
Oposs    ------------------------------------------------------------  1
Chick    ------------------------------------------------------------  1
Danio    SDLDEVIDSLFKEILTLD--EAAKLQDSQLEEIAIQLWNWAVTKRVGTSITEEQKAKVRH  79
Tetra    ------------------------------------------------------------  1
Branch   QRAETLSELLDKVSGLHGDSDKNRTSSGELQNCAVNLWNIAVAKRAGGHLSTGLNARLRH  120
Coral    ------------------------------------------------------------  1
Nemat    ------------------------------------------------------------  1

                                            W117R                   V142I
Human    VACKLLSMCEASFASEQSIQRLIMMNMRIGKEWLDAGNFLIADECFQAAVASLEQLYVKL  144
Mouse    IACKLVYMHGISVSSEEAIQRQILMNIKTGKEWLYTGNAQIADEFFQAAMTDLERLYVRL  145
Macaq    VACKLLSMCEASFASEQGIQRLIMMNIRIGREWLDVGNFLIADECFQAAVASLEQLYIKL  100
Dog      VACKLVCMCGGSAASEEAIRRQILMNMKTGKGWVDVGNAVIADEFFQAAMAGLEQLYVKL  164
Horse    VACKLVCMCEDSAVSEEAIQKQILMNMKTGKGWLDVGNAVTADEFFQVAIAGLEQLYVRL  133
Cow      VACKLIRMCEHPDASEEAIHRQILMNMKTGKGWVNVGNVVLADDFFQSAITSLEKLYSKI  141
Rat      IACKLVCMHGVSVSSEEAIQRQILMNMKTGKQWLDTGNAQIADEFFQAAMANLEKLYSRL  127
Oposs    ------------------------------------------------------------  1
Chick    ------MQCGVARRETSMRSAVPARSPEQAARMRPAVLSLSSGRAVLRDGLGHELLRELL  54
Danio    VACRLLYSCGPENPSESIVRKQILMASKTGRTWLDCKNSKSADAFLSMAVNSLETLYSRL  139
Tetra    ----------------------------------------------FSSYLQSLETLYSKL  15
Branch   LACQLAFKVSPLEGSEVVLKRRVMMASKTGRAWLDGGNPSMADNSLSLASECLEKLTKSV  180
Coral    ------------------------------------------------------------  1
Nemat    ------------------------------------------------------------  1

                                          Q172R
Human    IQRS----SPEADLTMEKITVESDHFRVLSYQAESAVAQGDFQRASMCVLQCKDMLMRLP  200
Mouse    MQSC----YTEANVCVYKMIVEKGIFHVLSYQAESAVAQGDFKKASLCVLRCKDMLMRLP  201
Macaq    IQRS----SPEADLIMEKITVERDHFRVLSYQAESAVAQGDFQRASMCVLQCKDMLMRLP  156
Dog      MQRS----STEIHMALHKIAMERDLFKVLSYQAEAAVAQGDFHRAYTCTLRCKDMLMRFP  220
Horse    MQRS----STEAHVAMQKIAVERDLFKVLSYQAESAVAQGDFQRASACVLRCKDVLMRVP  189
Cow      TQRS----PTEEHVMVQKNTVERDLLKVLSYQAEAAVAGGNFQKASRCILRCKDMLVRLP  197
Rat      MQSC----HTETNLSLYKNTVERLHCRYVNWEIETAVAQGDFKKASICVLRCKDMLIRIP  183
Oposs    ------------------------------------------------------------  1
Chick    REEA----PPRIAALTEELFQAAGALCEADGSAAEAVAQGDFQKAMQCVQRCKDMLVRLP  110
Danio    TSHG----DGE-DMNTPKGDIEKDLLRILSYQAESAVSQEQHQVAVSCIQRCKEILLRLP  194
Tetra    TSRT----DGSYDSTLSKEDVEKDLLRVLSFQAESALCQGNNAEALTCIQRCKDMLLRLP  71
Branch   LSAAAEGKLSEDDKERQKTEIEKDMFKVFCYQAESAVAQEQHDVAMRLAQRCKEMLARLP  240
Coral    ------------------------------------------------------------  1
Nemat    ----------------------------------AMSLGNNNSALECIEKAKAFLVKLP  25
```

```
                                                      T244I
Human     QMTSSLHHLCYNFGVETQKNNKYEESSFWLSQSYDIGKMDKK-STGPEMLAKVLRLLATN  259
Mouse     NMTKYLHVLCYNLGIEASKRNKYKESSFWLGQSYEIGKMDRR-SVEPQMLAKTLRLLATI  260
Macaq     QMTSSLHHLCYNFGVGTQKNNKYEESSFWLSQSYDIGKMDKK-STRPETLAKVLRLLATN  215
Dog       KMTGYLHILCYNFGVETHKQNKYEESSFWLSQSYDIGKMDKN-SVGPEMLAKVLRLLATA  279
Horse     KKTRYLHILCYNFGVETYKQNKYEESSFWLSQSYDIGKMEKN-SIGPEMLAKVLRLLATA  248
Cow       QMVCYLHTVCYNFGLQTYRENKYEESCFWLSQSFDIGKTDGN-SVEPEMLAKVLRLLATA  256
Rat       EMTKYLHVLCYNFGVESSKQNKYRESSFWLSQSYDIGKMGKD-SVEPEMLAKGLRLLATI  242
Oposs     ------------------------------------------------------------  1
Chick     RETRYLAILCYNFGVETYDCKKYEQSSFWLSQSYDIGKMDMKYSIGKEMQAKVLRLLATA  170
Danio     KETGYLSLICYNFGVDTYSQGKHEESTFWLSQSYDIGKMNMKYSPGSEMQAKVLRLLATV  254
Tetra     KDTAYLSLMCYNFGVDTYNMRKFEDSAIWLSQSYDIGKINVKYAPGSEIQAKILRLLATV  131
Branch    KETTFLAMLCYNFGVETYQKQKYEEAVAWLR-----------------------------  271
Coral     ------------------------------------------------------------  1
Nemat     KENATLSRLCYNFGVTTYNRREYEDCIEWLRESLELGKGKAP--VGHKKQATTLRLIANA  83

Human     YLDWDDTKYYDKALNAVNLANKEHLSSPGLFLKMKILLKGETSNEE--LLEAVMEILHLD  317
Mouse     YLNCGGEAYYTKAFIAILIANKEHLHPAGLFLKMRILMKGNSCNEE--LLEAAKEILYLA  318
Macaq     YLDWDNTKYYDKALNAVNLANKEHLNSTGLFLKMKILLKGETSNEE--LLEAVMEILHLD  273
Dog       YVDWDDQEYYDKALSAINLANKEHLNPSGLFFKMKILLKSERANEE--LLEAVMEILHLD  337
Horse     YLDWDDREYYDKALSAINLANKEHSHPAGLFLKMKILLKGEIANEE--LLEAVMETLHLD  306
Cow       YLDWDSSEYRDKALNIINLANKEHLDPVGLFLKVKILLKGEIGNEE--LFQNVMEILHLG  314
Rat       YLNCDDEAYYNKALIAVIVANKEHLDPAGLFLKMRILIKGKAFNEE--LLEAAKEVLYLA  300
Oposs     ------------------------------------------------------------  1
Chick     YFEWDCSLYLDKALKAINLANEENLHPAGFFLKVKILLKSGASDEG--ISSAVAEFQHHE  228
Danio     YLEWDHQKYLEKALQAVSLANKEHMQLSGLYLKIRILVKGCSPDEA--VKSGLSELLDCE  312
Tetra     YLEWDCQRFQEKALNAVNLANKECMSTSGLYLKIRILQTCGASDDD--IRAGLNEILEAE  189
Branch    ---------LTANLIFTGLANSEHSHPAGLHLKVHILLVSDTVEDNRLVSAVTDCLHHPE  322
Coral     ------------------------------------------------------------  1
Nemat     YLKWDS------------LANAEHPHPAGHYLKIELLLLSDNTPMARLQSAFEEALSLPD  131

Human     MPLDFCLNIAKLLMDHERESVGFHFLTIIHERFKSSENIGKVLILHTDMLLQRKEELLAK  377
Mouse     MPLEFYLSIIQFLIDNKRESVGFRFLRIISDNFKSPEDRKRILLFYIDTLLQKDQDMIAE  378
Macaq     MPLDFCLNIAKLLMDHEGESVGFHFLTIIHERFKSSENTGKVLLLYIDMLLQRKEELLAK  333
Dog       MSLDFCLNVAKLLMDHERESVGFYFMKIICEHFKSSENIGKALLLYIDMLLQRKEELLAK  397
Horse     ASLDVCLNIAKLLMDHDRESVGFYFLKIICEHFKSSENIGKALLLHIDMLLERKEELLAK  366
Cow       MTMNFCLNIVKLLMDHERDSVGFYFLKIICEHFQSSEDTSKAQLLHIDMLLQRKEDLFAK  374
Rat       MPLEFCLSIIQFLIDNKRDSVGFCFLKIIADHFKLPEHRKRILLFYIDMLLQVDQDVIAE  360
Oposs     ----MCL--------LARDSVGFDFLKTVCDRFESSADVGKGFLLHVKLLLQRKEDQLAK  48
Chick     MSLDFCLNTAKLLLEHGRESVGFDFLKSVAERFEASPDFGKVTLLYIEFLLQNKRELLAK  288
Danio     VPLEVCLSTVKLLVEENREALAFDFLKRVCQHFESSPELGSALLMHIELLLQRDKELLAK  372
Tetra     AALEECLSTVNLLMSEDREVLAFEFLKRVCQHFEASPDLGTALVLHAELLLQRGKELLAK  249
Branch    LTVELALNTIKLLIEHKRAQLALDGVKQLTQQFQASPELGKVYLLHLRILLDNRQMQAAK  382
Coral     ------------------------------------------------------------  1
Nemat     LTAQIGLSLAQLATRYKRTDLALNCLQTLATRFKNSPDLAKIQLQHVELLLQNNQDQEAK  191
```

```
Human     EKIEEIFLAHQTGRQLTAESMNWLHNILWRQAASSFEVQNYTDALQWYYYSLRFYST-DE  436
Mouse     EKIKDVLKGYQTRSRLSRDLVNWLHNILWGKASRSVKVQKYADALHWYSYSLKLYEY-DK  437
Macaq     EKIEEIILAHQTGRQLTAELKNWLHNILWRKATSSFEVQNYADALQWYYYSLRFYSP-DE  392
Dog       EKIDEIIIGQRTGRQLTTELVVCLHNILWKKAARSFEVQNYADALNWYYYSLRFYAT-DR  456
Horse     EKIEEIIVGHQTGRQLTTELVGCLHDILWKKAARSFEVQNYPDALQWYCYSLRLYAS-DQ  425
Cow       EKVEEIIIGHQTGKPLASELIGYFHNIIWKQAAKSFEVHNFDDALHWYCYSLRFYAA-DQ  433
Rat       EKIKEILIDHQTRSRLTRALVNWLHNILWGKATRCVKVQNYADALRWYSYSLKLYEC-DQ  419
Oposs     KKVEDILTAHHAGKELPPQILSWLHTILWDRAAQNFEAQNYFEALQWYNYSLNFYPS-GQ  107
Chick     QKVEEIIIGHYTGKQLLPETLNRLHIILWDTAAKHYEAKSYSEALHWYNYSVSFYTP-GQ  347
Danio     QKIEDAITGHYTGKQLASQTLSSLHLLLWDRASKNFEAKNFSEALQWYNYSLSFYKA-GE  431
Tetra     QKIEDIITDHYTGKQLSPQALTCLHVMLWDKACKYFEARNYPEALQWYNYSLSFFKA-GQ  308
Branch    ALVEDCITGHNTTQPLDTTTRKRFHLLLWEQAAQAYEANEYEEALRWYDYSRNLFSSNDR  442
Coral     ------------------------------------------------------------  1
Nemat     DLVEDCITGHNTGQQLSPDICKMFHLLLWEKAAN--------------------------  225

Human     MDLDFTKLQRNMACCYLNLQQLDKAKEAVAEAERHDPRNVFTQFYIFKIAVIEGNSERAL  496
Mouse     ADLDLIKLKRNMVSCYLSLKQLDKAKEAIAEVEQKDPTHVFTRYYIFKIAIMEGDAFRAL  497
Macaq     MDLDFAKLQRNMACCYLNLQQLDKAKEAVAEAERHDPRNVFTQFYIFKIAVIEGNSERAL  452
Dog       TDLDLAKLRRNMASCYLHLKQLDKAKEAVIEAEQRDPTNIFTQFYVFKIAVLEGNSGRAL  516
Horse     MDLDLAKLQRNMAACYLHLKQLDKAKVAVAEAEQRDPTNIFTQFYVFKIAVLEGNSDRAL  485
Cow       SDLDLAKLQRNMASCYLHLGQFDKAKDAVIKAAQRDPRNIFTQFYIFKISIVEGNSGRAL  493
Rat       ADMDLVKLKRNMVSCYLSLKQLDKAKEALAEAEQQDPTNIFTQYYIFKIAILDGEAYRAL  479
Oposs     MDADFAKLQRNRASCYIHLKQLEKANAAVKEAERFDAANIFTQFIIFKIAVLRDNTEEAL  167
Chick     IDQNLAKLQRNMASCYLHLKQIDKAKEAVKEAERCDLNSIFTQFSVYKIAVMENDTDKAV  407
Danio     LDPNLAKLQRNRSSCFLHLQQLDKAKEAVEEAARIDSANIFTQFNIYKIAILENNAEKAA  491
Tetra     LDPNLAKLQRNRVSCFLQLKQLEKAKDAVKEAQRSDPDSIFTHFSVYKVAVLENNVERAA  368
Branch    HDNNMAKLERNRSACYLHLKQYDKALEAARQAETCDPTSAHTQYALFKIGLLQGNSDAAI  502
Coral     ------------------------------------------------------------  1
Nemat     ------LYERNKCSCYTMLQDLKNASEAAAEAQRIEPNSPIVHFFQFKIALMKGDDNKAM  279

Human     QAIITLENILTDEESEDNDLVAERGSPTMLLSLAAQFALENGQQIVAEKALEYLAQHSED  556
Mouse     QVVSALKKSLMDGESEDRGLIEAGVSTLTILSLSIDFALENGQQFVAERALEYLCQLSKD  557
Macaq     QAIITLENVLTDEESEDNDLVAERGSPTMLLSLAAQFALENGQQIVAEKALEYLAQHSED  512
Dog       QAITTLEKLLE-EDPKENELLTDRDSPVMLLSIAAHFALENGQQIVAGKALEYLAQYSED  575
Horse     QAITSLEHLLTAEEPEENNLLTDRGSTVMLLSLAAQFALEHGQQIVAGKALEYLAQYSED  545
Cow       QAIFSLEYLLTTEEQNENNVSTKRTSGVMLFCLAAQFALENGQQVVAVKALENLAKYSEE  553
Rat       QIVSALKISMEE-DSEDCGLIENGVSTLRILSLCVEFALENEQQFVAQKALEYLIQLSKD  538
Oposs     TAVDALEKSVGNSAAQEKEMVKEENSSTAFLTFAAQFALENGQQDVARRALEYLSQHSQD  227
Chick     EAIIEMGKLAEKQSQHEDKLIVDESTSTNLLSLAAHIALENDQQVVAVRALEHLSEHSQD  467
Danio     AALRGIGILAKAPVSSEDRLLVTENAAANLLSLAAQIALEHEQQETAIKALETLCEHSED  551
Tetra     EALDAIGLLCKAPVSSEDRLLVSEDAASHLLKLAAQMALENEQQETAMKALESLCESSKD  428
Branch    EAIEKMGTVDEKGEENS--------TIEGLVCLAAQLAFEESNRDVAIRALERLVQHSHN  554
Coral     ------------------------------------------------------------  1
Nemat     EAIKQISQSQAN-DHDNNDVTTDEDDAHGLICLAAQLSLEQNNRSVAVKALDGILETSAS  338
```

```
Human    QEQVLTAVKCLLRFLLPKIAEMPES------------------------EDKKKEMDRLL  592
Mouse    PKEVLGGLKCLMRIILPQAFHMPES------------------------EYKKKEMGRLW  593
Macaq    QEQVLTAIKCLLRVVLPKIAEMPES------------------------EDKKKEMDRLL  548
Dog      PQQVLTALKCLFCLVFPRVSQMPES------------------------ENKKKEMDRLL  611
Horse    PQQVLTALKCLFRLVLPKVSQMPES------------------------ENKKKEMDRLL  581
Cow      PPQVLTALKCLFRLCLPRISQMPES------------------------ENKRQEMDRIL  589
Rat      PREVLEALKCLVRIILPQAFYLPES------------------------EIKKKEMNRLL  574
Oposs    LQQVLTALKCLIRLTVPQISQTTECEGNRSDLFLETLFASPFILRVVPLKRRRVKFSSMS  287
Chick    CQQVFAALKCLVRLTLSKVEKE---------------------------EKRDNDIKSML  500
Danio    VPQTLTALRCLVRLALSTLENISE-------------------------ENRNASLDILL  586
Tetra    AGLVLSALRCLVRLVLSATEQLTE-------------------------GMRARGLVQED  463
Branch   IQQVLTAIRSSNDIILS-------------------------------------------  571
Coral    ------------------------------------------------------------  1
Nemat    NKQVITALRCLIRLKLTISETDVKG----------------------DVNGIMPYLKKAL  376

               Exon 22-encoded aa sequence
Human    TCLNRAFVKLSQPFGEEALSLESRANEAQWFRKTAWNLAVQCDKDPVMMREFFILSYK--  650
Mouse    NYLNTALLKFSEYFNEAPSTLDYMVNDANWFRKIAWNLAVQSEKDLEAMKNFFMVSYK--  651
Macaq    TCLNRAFVKLSQPFGEEDLSLESRVNEAQWFRKIAWNLAVQCDKDPVIMREFFILSYK--  606
Dog      TCLNTALQKLGQSLNGEALNSDSRAKEAHWFRKIAWNLAVQCGKDLVTMREFFILSYK--  669
Horse    TCLNIALLKLAQFFDGKASTSDSRTNEAHWFRKIAWNLAVQCDKDPVTMREFFMLSYK--  639
Cow      AYFNTALLNLTQLFEGETWTLASKINEAHWFRKTAWNLAVQCEKDPLSMREFFVLSYK--  647
Rat      SYLDTALLKFSQHFDDTSSTLDPMVNDANWFRKIAWNLALQSEKDLEKMKNFFMISYE--  632
Oposs    NKLEIAHQRLAEPVAKENLTLDVWTNEAHWFRKIAWNLAMQCEKCPGTMRDFFLLSYE--  345
Chick    TYLTLAHQRLAEPFTEENLTRDIRTSEAHWFRKVAWNLAVQLKDCPEKMRDFFVLSFK--  558
Danio    SYLKTALQKLSQVCHIPGQRAEQRSEDANWFRRIAWNSALQCEHSPVRMKDFFLLSFQ--  644
Tetra    RYQR-ALPRLSLNSLLHSDKPS--------FCAPAWNSALYCEKCPDRMRDFFVLSYQAS  514
Branch   ------------------------------YLETAWNLGLKCGEDTNMMHQFFNMCFK--  599
Coral    ------------------------------------------------------------  1
Nemat    ECVNKMAALGPLADVQAEATWFMKIGNTILQKLAPWNMALDSSESSSDLREFFLICYK--  434


Human    MSQFCPSDQVILIARKTCLLMAVAVDLEQGRKASTAFEQTMFLSRALEEIQTCNDIHNFL  710
Mouse    LSLFCPLDQGLLIAQKTCLLVAAAVDLDRGRKAPTICEQNMLLRTALEQIKKCKKVWNLL  711
Macaq    MSQFCPSDQVILIARKTCLLMAAAVDLEQGRKASTAFEQTMFLSRALEEIQTCNDIQNFL  666
Dog      LSQFCPSDQVILIAQKTCLLMAAAVDLEQGRKASTAFEQTRLLNRALEQIHKCRHTWNLL  729
Horse    LSQFCPSDQVILIAQKTCLLMAAAVDLEQGRKASTTFQQVMLLNRALEQIHKCRDIWNLL  699
Cow      MSLFCPSDQVILIAQKTCLLMAAAVDLQQGRRASTAFEQNCFLNRALDLIRKCRDIWNVL  707
Rat      LSLFCPSDQGLLIAQKTCLLVAAAVDLEQGRKATTTYEQNKLLRMALEQIQKCKNVWNLL  692
Oposs    LSQLCPSEKTVLTSQKACLLMVAAADLELGRSASEASQQTELLSRALEHIQGCKEISNVL  405
Chick    LSQFCPSDKAVLIAQKTCLLMAAAIDLELGRQEVTPSEQVEFWNQALQHLQACKEIWKVL  618
Danio    LSQLCAPDRAVLMGQKTCLLMSAAASLEICRSSDHTEQQTELLTQTLENIQLCKEIWTTI  704
Tetra    LSQLCPPDRGLLTGQKTCLLMAAAASLELCRKS-PLSDQTEELTRVLEQIQTCWEVWKTL  573
Branch   FSSLCPMDMANLVRKKTCALMAAAACLQSARNVSDPGEKMEMLKTVILHVDACRETCREI  659
Coral    ------------------------------------------------------------  1
Nemat    ---------------------------------------KEALSETLTHVQNCRDLCKRI  455
```

```
                                                    V748A
Human     KQTGTFSNDSCEK-----LLLLYEFEVRAKLN--DPLLESFLESVWELPHLETKTFETIA  763
Mouse     KKTGDFSGDDCGV-----LLLLYEFEVKTKTN--DPSLSRFVDSVWKMPDLECRTLETMA  764
Macaq     KQTGTFSNDSCEK-----LLLLYEFEVRAKLN--DPLLESFLESVWELPHLETKTFETIA  719
Dog       KETGDFSSDPCET-----LLLLYEFEVKAKMN--DPLLDSFLESVWELPHLESKTFETIA  782
Horse     KETGDFSNDPCET-----LLLLYEFEVKAKMN--DPFLDSFLESVWELPHLESKTFETIA  752
Cow       KTTGDFSRDPCET-----LLILYEFEVRAKLN--DPLLDHFLEPVWELPTLESKAFETMA  760
Rat       KQTGDFSGDDCGV-----MLLLYEFEVKTKTN--DPSLHSFLQSVWKMPGLESRTLEIMA  745
Oposs     KLAGDGSKDPTEN-----LLLLYEIEARAKLH--DAGLTSLLESVWELPQLETKILETIA  458
Chick     KLTGDFSKDPTDT-----LLLLYEFEARSKLN--DPTLNNFMESVWEQP-HEIKTLEIVA  670
Danio     KTSGISSQDKTDS-----LLLLYEFEARAKLN--DPKLETVLESVLELDNIETKLLEVIA  757
Tetra     KASGNLSVDPTDTL----LLLLYEFEARAKLN--DPKVETVLEAVLELQNVEPKVLETIA  627
Branch    CNSKITDTDCSGDST-PILLCLYEFEAKAKLG--EPDLTQLVDRVMAMPQADAKTLETMA  716
Coral     -----------------------------HLG--DNNLSECLDMVEAMPHTDPNTFETLA  29
Nemat     NGTHSIQGNKDKKDDSIILLILYEFEAKAKLGEGDVSLESCLHKLIALPFCEAKTFETVA  515
                                         :    :  :   :. :      : . :* :*

Human     IIAMEKPAH------------YPLIALKALKKALLLYKKEEPIDISQ-------------  798
Mouse     LLAMDKPAY------------YPTIAHKAMKKLLLMYRKQEPVDVLK-------------  799
Macaq     IIAMEKPAH------------YPFIALKALKKALLLYKKEESIDVSQ-------------  754
Dog       SLAVEMPAH------------YPSIALKALKEALLLYKKKESIDVLK-------------  817
Horse     SLAMEMPAC------------YPSIALKALKRALLLHKRKESIDVLK-------------  787
Cow       ALAMEMPAC------------YPSIALKALKKALFFYKRKESIDVVR-------------  795
Rat       SLAMDKPAH------------YPTIAQKALKKLLLIYRRKVPINVLR-------------  780
Oposs     SVAMEHPAH------------YPDIAKKALRKALSLYLQGESIDVAK-------------  493
Chick     SLAMEPPAR------------YPVLCKKALKSALNLYRKETTTDAVK-------------  705
Danio     VLAMEPPAH------------YPVLCKKALKIALSLHRKQPQVDLMR-------------  792
Tetra     ALAMEPPAH------------FPRLCKKALRVALALHRKQPQADLTR-------------  662
Branch    AVCC--------------------------------------------------------  720
Coral     ALAVESPVY------------HRDLSMRSLRIAIKKCLAMDVIDFTK-------------  64
Nemat     GITNNEPFFPVIVWIALFAGPPRGVSMLIQDLLEEGKLALHKIIFTRNNYFALPVHTFDF  575
           :

Human     -YSKCMHNLVNLSVPDGASNVELCPLEEVWGYFEDALSHISRT-KDYPEMEILWLMVKSW  856
Mouse     -YSVCMHNLIKLLVADEVWNISLYPLKEVQSHFKNTLSIIRQN-EGYPEEEIVWLMIKSW  857
Macaq     -YSKCMHNLINLSVPDGASNVELCPLEEVWGYFEDALSHISHT-KDYPEMEILWLMVKSW  812
Dog       -YSKCMHNLINLLVPDGVPSTELCPLEEVWGYFEDALSLISHT-KGYPETEILWLMIKSW  875
Horse     -YSKCMHNLINLLVPERVLSAELCPLEEVWGYFEDALHLISHT-EGYPETEILWLMVKSW  845
Cow       -YSQCMHSLVNLLVPDGVPNTELCPMEEIWGNFEDALIFISQT-EGYPEMEVLCLMIKSW  853
Rat       -YSVCMHNLIDLLVSDRRWNMALYPLKEVWGHLKSALSLIRQT-KGYPEEEIVWLMIKAW  838
Oposs     -FSKCLHSFINLSLPDEALSTDVCSLQDIWSTFEVALRVISHS-EEYPQVEILWLMTKAW  551
Chick     -FSKCLHSLINLSLPTGVTDLDACVLQEVWGYFEDALSVVSST-DSYPEMEILWLMTRAW  763
Danio     -CSNCLHSLIQLSLPNGVSEVQPCVLEEVWGYYEEALSLIATT-EDFPELEILWLLTRAW  850
Tetra     -CSKCVHSLIKLSLPSGVSEVEARVLEEVWEYYEEALSMIAASPDDFPEMETLWLLTRAW  721
Branch    ------------------------------------------------------------  720
Coral     -LSKAFHSL---------------------------------------------------  72
Nemat     MFSKVFHSLAELALGRGSSRDAESKEEAWTLYQEIIEFVESTDKGSYPEMELIWLMTKAW  635
```

```
Human     NTGVLMFSRSKYASAEKWCGLALRFLNHLTSFKESYETQ-------MNMLYSQ-------  902
Mouse     NIGILMSSKNKYISAERWAAMALDFLGHLSTLKTSYEAK-------VNLLYAN-------  903
Macaq     NTGILMFSRSKYVSTEKWCGLALRFLDHLTSFKESYETQ-------MNMLYSQ-------  858
Dog       NTGIFMYGRNKYVSAEKWCGLALRFLDRLGSLKRSYETQ-------MNILYSE-------  921
Horse     NIGIFMYGSSKYVSAEKWCSLALRFLDHLGSLRRSYEIQ-------MNVLYSQ-------  891
Cow       NIGIFLYSERMYVSTEKWCGLALRLLDYLGSLKSTYETQ-------VNILYGE-------  899
Rat       NIGILMYSRNKYISAERWARMTLEFLDHLGSLKTNYEAKHPFRLLPTSCLHAVSHSQKYL  898
Oposs     NTGVSQYKEGMYATAEKWCHLGIRFLDHLGSLKKCYEGQ-------MADLYGE-------  597
Chick     NTGIFQYTVGKYQEAEQWCGLGMRFLNHLGSLKRSYEGH-------VSQMPES-------  809
Danio     NTGILLYSLAQYPEAERWCGLGMSFLRYLGSLQDSYQTQ-------MAGLYSE-------  896
Tetra     NTGILLYSLAPYPAAERWCGLAMSFVRHLGSLQQSYGTQ-------VNS-----------  763
Branch    ------------------------------------------------------------  720
Coral     ------------------------------------------------------------  72
Nemat     NCGINLFSSGRFDASEKWCATAMRLLQRLVGFKTNYESK-------MTSVYSD-------  681

Human     LVEALSNNKGPVFHEHGYWSKSD-------------------------------------  925
Mouse     LMEILDKKTD------------------------------LRSTEMTEQ-----LRALIV  928
Macaq     LVEALSNNKGPVFHEHGYWSKSD-------------------------------------  881
Dog       LVEALDRKKGSLFNEE--------------------------------------------  937
Horse     LVEALDKNKGSLFNKECPLETEDRSQAPPCPLGFFFFRLNLLRPHTCSSQDVVTSPSKSE  951
Cow       LMEALEKNKRSAFNEE--------------------------------------------  915
Rat       LQALLKGHKDDWTSPGWTHPTFTVGKVAGMAALQGQQLLSVKSPSSSSQPKPLLLRCRLA  958
Oposs     VLTKVERDKSLPPNEE--------------------------------------------  613
Chick     ------------------------------------------------------------  809
Danio     VLDRLDKAKKNLIIEE--------------------------------------------  912
Tetra     ------------------------------------------------------------  763
Branch    ------------------------------------------------------------  720
Coral     ------------------------------------------------------------  72
Nemat     ILDRISRAAAQATVEE--------------------------------------------  697

Human     ------------------------------------------------------------  925
Mouse     PP------EDQGSVSSTNVAAQN-----HL------------------------------  947
Macaq     ------------------------------------------------------------  881
Dog       ------------------------------------------------------------  937
Horse     CRWQKRDRTSRWFIQSNVKTTSGYPMAPTGTALQIKSSWGFTAEAARLFSGKAHGIETLF
1011
Cow       ------------------------------------------------------------  915
Rat       PPGANLSSEENDQIQSCKPAVQGRNAQGHLITWAVSLTSQAVQLSRVCLSVSPRLFSALL
1018
Oposs     ------------------------------------------------------------  613
Chick     ------------------------------------------------------------  809
Danio     ------------------------------------------------------------  912
Tetra     ------------------------------------------------------------  763
Branch    ------------------------------------------------------------  720
Coral     ------------------------------------------------------------  72
```

```
Nemat     ------------------------------------------------------------  697


Human     -------------------------------  925
Mouse     -------------------------------  947
Macaq     -------------------------------  881
Dog       -------------------------------  937
Horse     AEGGTRCAGASSAISCK--------------  1028
Cow       -------------------------------  915
Rat       LGVYENNNSDGFAPVLARERSMQEWARSHDE  1049
Oposs     -------------------------------  613
Chick     -------------------------------  809
Danio     -------------------------------  912
Tetra     -------------------------------  763
Branch    -------------------------------  720
Coral     -------------------------------  72
Nemat     -------------------------------  697
```

Supplementary Figure 2

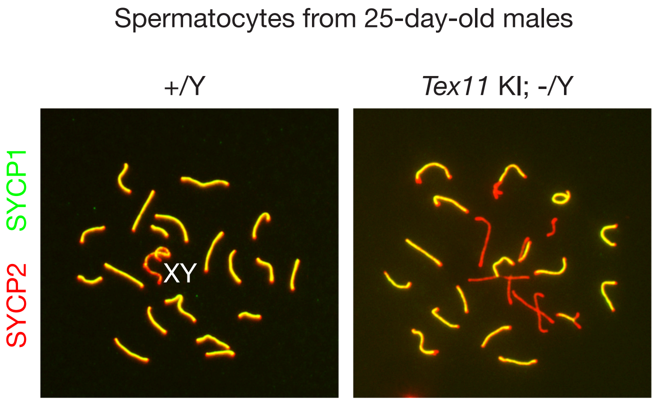

Supplementary Figure 3

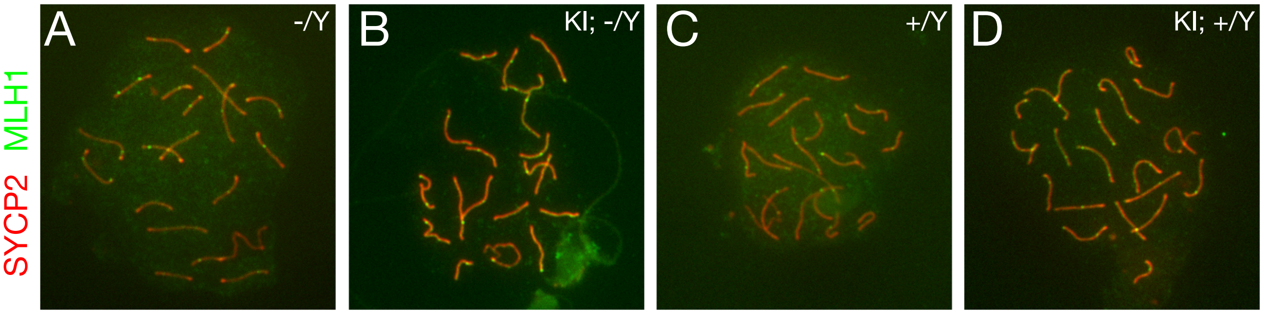

Supplementary Figure 4

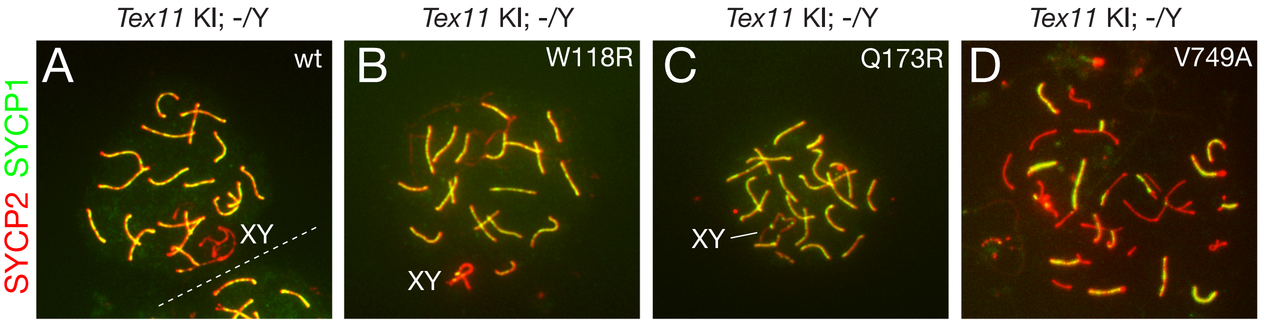

Supplementary Figure 5

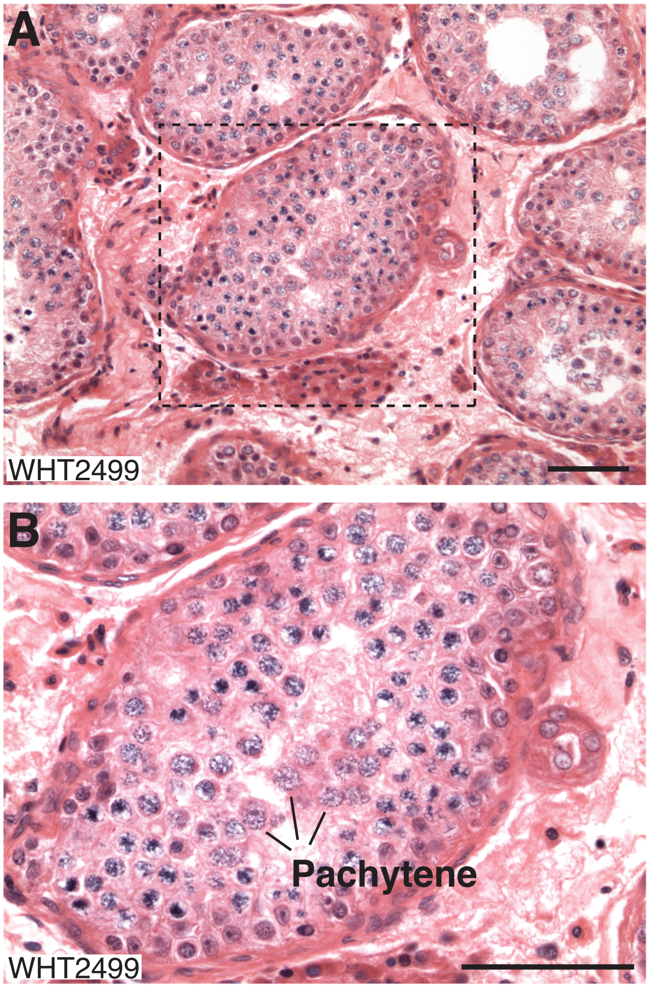

Supplement: Supplementary file 1 [file emmm0007-1198-sd1.pdf]
