# Supplementary material for: TEX11 is mutated in infertile men with azoospermia and regulates genome-wide recombination rates in mouse
Source: EMBO Mol Med. 2015 Jul 1;7(9):1198–210. doi: 10.15252/emmm.201404967 (PMC4568952; doi:10.15252/emmm.201404967)
Supplement: Supplementary file 2 [file emmm0007-1198-sd2.pdf]

EMBO Molecular Medicine

TEX11 is mutated in infertile men with azoospermia and regulates genome-wide recombination rates in mouse

Fang Yang, Sherman Silber, N. Adrian Leu, Robert D. Oates, Janet D. Marszalek, Helen Skaletsky, Laura G. Brown, Steve Rozen, David C. Page, P. Jeremy Wang

Corresponding author: P. Jeremy Wang, University of Pennsylvania

Review timeline:

Transfer date:	11 December 2014
Editorial Decision:	14 January 2015
Revision received:	13 May 2015
Editorial Decision:	03 June 2015
Revision received:	03 June 2015
Accepted:	05 June 2015

Transaction Report:

(Note: With the exception of the correction of typographical or spelling errors that could be a source of ambiguity, letters and reports are not edited. The original formatting of letters and referee reports may not be reflected in this compilation.)

Editor: Céline Carret

1st Editorial Decision 14 January 2015

Thank you for the submission of your manuscript to EMBO Molecular Medicine. We have now heard back from the two referees whom we asked to evaluate your manuscript.

As you will see, both referees are enthusiastic about your study but still raise a few issues that have to be addressed in a revised version of your article. We would like to particularly encourage you to experimentally address the point 5 made by referee 2 as we agree this would strengthen the main findings. Regarding the other comments, you will see that they relate to the structure/organization and wording of the text and would appreciate if you could address them to improve clarity and readability.

Therefore, we are happy to invite you to submit a revised version for further consideration and depending on the nature of the revisions, this may be sent back to the referees for another round of review.

Please read below for important editorial formatting.

I look forward to seeing a revised form of your manuscript as soon as possible.

***** Reviewer's comments *****

Referee #2 (Comments on Novelty/Model System):

This manuscript shows the power of combining good genetic analyses of human diseases with targeted knockout mouse models.

Referee #2 (Remarks):

The manuscript of Yang et al is well written and complete. I do have a few comments and concerns however, which should be addressed prior to publication.

1) In general, there is quite a bit of introduction and discussion placed into the results section. While this does help the authors frame their experiments, several paragraphs on importance of results would be better placed in the discussion.

2) Please note that all bands for Western blot images are missing from Figures provided for review.

3) For quantified data from immunoflorescent images, please provide example images.

4) Methods section needs to be greatly expanded with citations for method validation and software packages used to quantify and analyze data.

5) While spermatogenesis may be rescued with the knock in approach the authors employed, analysis of the fertility potential -i.e. are these sperm capable of fertilizing an oocyte and normal embryogenesis - should be evaluated. At the minimum, a fertility trial of these knock-in mice should be performed. And, the same goes for the increased numbers of MLH1 foci in oocytes: are these oocytes arrested or will they undergo normal embryogenesis upon fertilization?

Overall these are very good data with high translational importance.

Referee #3 (Comments on Novelty/Model System):

Novelty overall is medium because the knockout allele was characterized years ago and so the phenotype here is a refinement. The functional testing of alleles is nice and relatively novel. Medical impact....for a subset of affected patients, but nothing can be done....yet.

Mouse modeling is powerful with no ethical concerns

Referee #3 (Remarks):

This is an interesting manuscript that makes a strong case that mutations in the X-linked TEX11 gene underlying a significant fraction of males with non-obstructive azoospermia (a drastic form of infertility). In my opinion, this is the strongest aspect of the manuscript, in particular because the authors actually used a clever knockin system to functionally test 3 missense alleles in mice (finding that 1 of the three was a likely separation of function infertility allele). They also find that crossover numbers is related to the level of Tex11 expression. This paper will be of significant interest in the reproductive biology field. The data presented is sound.

The major shortcoming, in my opinion, is in the presentation of the paper, some parts are overhyped (and not that important in my mind), while other parts are underhyped (and more important in my mind!). The title doesn't mention the human infertility aspect, nor does it allude to the functional evaluation of alleles in transgenics - both of which are strong points of the paper (as is the nice little

family study of the truncation allele). Also, I think it very important that the authors not only obtained functional evidence for 1 missense allele being an infertility allele, but that they also disproved 2 alleles. This underscores the importance of such validation studies and distinguishes this paper as being a cut above papers that make no such valid attempts.

Indeed the authors find solid evidence for dosage of Tex11 affecting recombination, but the effect is relatively minor and non-linear (less than 2 MLH1 foci/spermatocyte difference from 1 to 2 doses of the gene), though significant. On its own, this part of the story would have to be more developed, because it does not actually deal with "recombination," but rather MLH1 foci...a proxy for chiasmata/crossovers. There is no discussion of the different pathways for crossing over (Mus81 dependent/independent) or the spacing of crossovers (interference vs non-interference pathways), or actual chiasmata counts. If the authors insist on highlighting this angle, I think such additional experiments are required. If not, the shortcomings ("future work") should be outlined.

Also, as I write below, the idea that the knockin allele provides the first evidence for X>autosome transposition of meiosis genes is overhyped and doesn't reflect well on the paper. So, overall, this is an important piece of work, and with re-writing, its importance will be of higher profile. Below is a list of recommendations for the manuscript.

- The abstract needs some work. The second sentence of the abstract doesn't make sense; it doesn't follow that the X is enriched for spermiogenesis genes because it is hemizygous. The phrase "....TEX11 mutations associate...." implies a GWAS. Please rephrase to reflect he actual study done. Also, I'm not sure it is appropriate to say that TEX11 is a "hotspot" for infertility mutations; rather, because it is on the X, mutations that arise will manifest as infertility and thus it is easier to detect such mutations. Finally in abstract, the sentence beginning "An autosomal..." isn't very clear. What is a "long-standing phenomenon"? I think it is an overstatement to say that because the transgene rescues the knockout (actually, only partially!), that this provides "genetic evidence" for X-to-autosome retrotransposition. The logic simply doesn't follow. Later in the Results it is stated that this experiment "provides the first direct genetic evidence that such a gene can substitute for the function of its X-linked ancestral gene." Yet, the KI/KO mice still have marked defects, which would seem to contradict this conclusion.

- The title of the paper, and also the section entitled, "TEX11 is a dosage-sensitive regulator of meiotic recombination levels" are slightly grammatically incorrect. They imply that what is dosage-sensitive is TEX11 itself. Somehow, these phrases should be re-written to indicate that recombination rates are sensitive to Tex11 dosage. Even the term "dosage" is somewhat unclear because it implies genetic dosage; meanwhile the data indicate that the levels of TEX11 protein are important.

- Given that a substantial fraction of Tex11 mutations seems benign, yet infertility patients have more of them (compared to controls), how do the authors interpret this? There are many reports in the literature of candidate gene studies finding more variants in the patient (infertile) group compared to controls, and most of those studies don't bother to functionally test the variants as done here. The authors might want to comment.

- Earlier in the Results, I suggest mentioning that the patients in the study were selected to not have Y microdeletions.

- Fig. 2: The term "X-to-autosome retrotransposition" is incorrect. I know the authors are pushing it as a model for this, but in fact this is a knockin allele.

1st Revision - authors' response 13 May 2015

Thank you for the two reviews of our manuscript. We have conducted additional experiments to address concern no. 5 raised by referee #2; these new data are presented in revised Fig 6 (new panel E). In response to the reviewers' suggestions, we have added three supplementary figures (Fig S2-4) and have made changes to the text as detailed below.

***** Reviewer's comments *****

Referee #2:

The manuscript of Yang et al is well written and complete. I do have a few comments and concerns however, which should be addressed prior to publication.

1) In general, there is quite a bit of introduction and discussion placed into the results section. While this does help the authors frame their experiments, several paragraphs on importance of results would be better placed in the discussion.

Response

We have improved the organization of the manuscript by moving some paragraphs from the results to the discussion. Specifically, the paragraph on the significance of X-to-autosomal retrotransposition has been moved to the discussion (last paragraph). The paragraph on the description of hurdles in the genetic studies of human male infertility has been moved to the introduction (2nd paragraph).

2) Please note that all bands for Western blot images are missing from Figures provided for review.

Response

We have verified that Western blot images were not lost after file conversion.

3) For quantified data from immunoflorescent images, please provide example images.

Response

The revised manuscript includes three new supplementary figures providing representative example images: chromosomal asynapsis in 25-day-old males (Supplementary Fig 2 – related to Fig 3B), number of MLH1 foci in 3-month-old males with different *Tex11* gene dosages (Supplementary Fig 3 – related to Fig 4A), and chromosomal synapsis in 3-month-old knockin males (Supplementary Fig 4 – related to Fig 6F).

4) Methods section needs to be greatly expanded with citations for method validation and software packages used to quantify and analyse data.

Response

We have expanded the Methods section and have added two new paragraphs: "Mating tests and sperm count" and "Statistics".

5) While spermatogenesis may be rescued with the knock in approach the authors employed, analysis of the fertility potential -i.e. are these sperm capable of fertilizing an oocyte and normal embryogenesis - should be evaluated. At the minimum, a fertility trial of these knock-in mice should be performed. And, the same goes for the increased numbers of MLH1 foci in oocytes: are these oocytes arrested or will they undergo normal embryogenesis upon fertilization?

Overall these are very good data with high translational importance.

Response

We have performed fertility trials revealing that knockin males of all genotypes were fertile and produced comparable litter size of pups. The pups were viable and appeared to be healthy. The sperm count in the KI(V749A)/KO males was significantly lower than controls, but was high enough ($1.7x10^7$) so that their fertility was not reduced. These new data are presented in a new panel – Fig 6E. For females, we examined the number of MLH1 foci in wild type, *Tex11*$^{+/-}$, and *Tex11*$^{-/-}$ ovaries (Fig 4B). Outcomes of mating tests of these females have been reported previously, showing that *Tex11*$^{-/-}$ females produce smaller litters than controls (Yang et al., 2008).

Referee #3:

This is an interesting manuscript that makes a strong case that mutations in the X-linked TEX11 gene underlying a significant fraction of males with non-obstructive azoospermia (a drastic form of infertility). In my opinion, this is the strongest aspect of the manuscript, in particular because the authors actually used a clever knockin system to functionally test 3 missense alleles in mice (finding that 1 of the three was a likely separation of function infertility allele). They also find that crossover numbers is related to the level of Tex11 expression. This paper will be of significant interest in the reproductive biology field. The data presented is sound.

The major shortcoming, in my opinion, is in the presentation of the paper, some parts are overhyped (and not that important in my mind), while other parts are underhyped (and more important in my mind!). The title doesn't mention the human infertility aspect, nor does it allude to the functional evaluation of alleles in transgenics - both of which are strong points of the paper (as is the nice little family study of the truncation allele). Also, I think it very important that the authors not only obtained functional evidence for 1 missense allele being an infertility allele, but that they also disproved 2 alleles. This underscores the importance of such validation studies and distinguishes this paper as being a cut above papers that make no such valid attempts.

Response

We have revised the title to include the human infertility aspect. The abstract and the text have also been revised as suggested to improve organization and clarity. Please see below our detailed response in response to this summary comment and individual remarks.

Indeed the authors find solid evidence for dosage of Tex11 affecting recombination, but the effect is relatively minor and non-linear (less than 2 MLH1 foci/spermatocyte difference from 1 to 2 doses of the gene), though significant. On its own, this part of the story would have to be more developed, because it does not actually deal with "recombination," but rather MLH1 foci...a proxy for chiasmata/crossovers. There is no discussion of the different pathways for crossing over (Mus81 dependent/independent) or the spacing of crossovers (interference vs non-interference pathways), or actual chiasmata counts. If the authors insist on highlighting this angle, I think such additional experiments are required. If not, the shortcomings ("future work") should be outlined.

Response

The non-linear effect of the *Tex11* gene dosage on the number of MLH1 foci is most likely due to crossover interference and crossover homeostasis. Current hypotheses suggest that these two mechanisms (which are of largely unknown molecular basis) regulate the wide spacing of crossovers on the same chromosome and maintain the number of crossovers relatively constant, respectively. Zip4, the yeast homologue of TEX11, belongs to the ZMM group proteins, which are involved in the Mus81-independent but MLH1-dependent crossover pathway. Disruption of TEX11 leads to a

significant reduction in the number of MLH1 foci in mice. Therefore, TEX11 functions in the MLH1-dependent pathway. We have discussed these points and outlined future work in a new paragraph – the third paragraph in the revised discussion.

Also, as I write below, the idea that the knockin allele provides the first evidence for X>autosome transposition of meiosis genes is overhyped and doesn't reflect well on the paper. So, overall, this is an important piece of work, and with re-writing, its importance will be of higher profile. Below is a list of recommendations for the manuscript.

- The abstract needs some work. The second sentence of the abstract doesn't make sense; it doesn't follow that the X is enriched for spermiogenesis genes because it is hemizygous. The phrase "....TEX11 mutations associate...." implies a GWAS. Please rephrase to reflect he actual study done. Also, I'm not sure it is appropriate to say that TEX11 is a "hotspot" for infertility mutations; rather, because it is on the X, mutations that arise will manifest as infertility and thus it is easier to detect such mutations. Finally in abstract, the sentence beginning "An autosomal..." isn't very clear. What is a "long-standing phenomenon"? I think it is an overstatement to say that because the transgene rescues the knockout (actually, only partially!), that this provides "genetic evidence" for X-to-autosome retrotransposition. The logic simply doesn't follow. Later in the Results it is stated that this experiment "provides the first direct genetic evidence that such a gene can substitute for the function of its X-linked ancestral gene." Yet, the KI/KO mice still have marked defects, which would seem to contradict this conclusion.

<u>Response</u>

We have revised the abstract extensively in response to these concerns. In particular, we removed “association” and “hotspot”. We agree that due to the X chromosomal location of *TEX11*, mutations that disrupt protein function will manifest as infertility and thus are readily detected; this understanding is reflected in the revised manuscript (introduction and discussion). There are a number of X-to-autosomal retrogenes. It has been hypothesized (but not been shown experimentally) that such autosomal copies compensate for the loss of function of the respective X-linked gene due to X chromosome silencing during male meiosis (i.e. MSCI). The mice generated in our study represent an ideal model to test this hypothesis, because they lack an X-linked gene that is essential for fertility but carry a transgenic copy of this gene on an autosome, which therefore resembles an X-to-autosomal “retrogene”. Our data show that the autosomal copy rescues the fertility of the X-linked KO. Indeed, the KI/KO mice still have marked defects at the juvenile age but are fertile at the adult age. It is very likely that a new retrogene was not functionally equivalent when it first appeared during evolution, but became functional over time. In summary, our study provides the first experimental model to test and support the hypothesis that X-to-autosomal retrotransposition evolved as a compensatory mechanism. We have modified the language to emphasize that the knockin represents a model for a retrogene.

- The title of the paper, and also the section entitled, "TEX11 is a dosage-sensitive regulator of meiotic recombination levels" are slightly grammatically incorrect. They imply that what is dosage-sensitive is TEX11 itself. Somehow, these phrases should be re-written to indicate that recombination rates are sensitive to Tex11 dosage. Even the term "dosage" is somewhat unclear because it implies genetic dosage; meanwhile the data indicate that the levels of TEX11 protein are important.

<u>Response</u>

We have revised the title accordingly. Our data show that levels of TEX11 protein correlate with *Tex11* gene dosage. In the revised manuscript, we refer to both gene dosage and protein levels.

- Given that a substantial fraction of Tex11 mutations seems benign, yet infertility patients have more of them (compared to controls), how do the authors interpret this? There are many reports in the literature of candidate gene studies finding more variants in the patient (infertile) group compared to controls, and most of those studies don't bother to functionally test the variants as done here. The authors might want to comment.

Response

It is very likely that more of the mutations identified in infertile men are causative. However, without functional evaluation as performed for the three missense mutations in this study, it is unclear whether they are benign or causal. Functional evaluation of human mutations in mouse models is expensive, time-consuming, and technically demanding. We think that this is the main reason that this has not been done before. The new CRISPR/Cas9 genome-editing technology will likely ameliorate these hurdles but is still costly using the mouse model.

- Earlier in the Results, I suggest mentioning that the patients in the study were selected to not have Y microdeletions.

Response

This information has been added.

- Fig. 2: The term "X-to-autosome retrotransposition" is incorrect. I know the authors are pushing it as a model for this, but in fact this is a knockin allele.

Response

The reviewer is correct that this is a knockin allele. However, because the *Tex11* knockin allele generated in this study lacks all 29 *Tex11* introns, it resembles a "retrotransposed" gene and can therefore serve as an experimental model to test the compensatory function of an X-to-autosomal retrogene (see also response above). Therefore, we have kept this description in this context.

2nd Editorial Decision 03 June 2015

Thank you for the submission of your revised manuscript to EMBO Molecular Medicine. We have now received the enclosed reports from the referees that were asked to re-assess it. As you will see the reviewers are now supportive and I am pleased to inform you that we will be able to accept your manuscript pending final editorial amendments.

***** Reviewer's comments *****

Referee #2 (Remarks):

Thank you for thoroughly and thoughtfully addressing all concerns.

Referee #3 (Comments on Novelty/Model System):

They were outlined in original review

Referee #3 (Remarks):

The authors have responded to the reviews well. I have no remaining issues with the excellent manuscript.
